# Beyond GMOs: transgene-free gene-edited crops for global food security

**DOI:** 10.3389/fpls.2026.1738485

**Published:** 2026-07-10

**Authors:** Aftab Ahmad, Muhammad Faheem, Annena Ijaz, Anam Niamat, Noor ul Huda, Ahmad Munir, Saqib Siddique, Sajjad Asaf, Hamad Khan, Nayla Munawar, Ahmed Al Harrasi

**Affiliations:** 1 Natural and Medical Sciences Research Center (NMSRC), University of Nizwa, Nizwa, Oman; 2 Department of Biochemistry, University of Agriculture, Faisalabad, Pakistan; 3 National Institute for Biotechnology and Genetic Engineering (NIBGE), Faisalabad, Pakistan; 4 Department of Chemistry, College of Science, United Arab Emirates University, Al-Ain, United Arab Emirates

**Keywords:** transgene-free gene-edited crops, CRISPR genome editing, precision breeding, genome editing regulation, food security, ethical considerations

## Abstract

Transgene-free gene-editing has transformed the genomic landscape of crops by enabling targeted, precise, and predictable genetic outcomes without integrating any foreign DNA into the host genome. It has significantly reduced production time and costs, and the regulatory burden for transgene-free gene-edited crops, while improving social acceptance compared with classical transgenic crops. This review compares the transgene-free gene-edited, transgenic, and cisgenic crops. We also focus on core methods for developing transgene-free gene-edited crops, particularly ribonucleoprotein (RNP), transient expression, the transgene killer method, and HI-edit technology. We highlight the practical examples summarizing CRISPR applications for transgene-free gene-edited crops, including cereals, legumes, and oilseeds, and horticultural crops. We also analyze the rapidly evolving global regulatory landscape of transgene-free gene-edited crops, including the recent European Union movement towards differentiated oversight for certain “new genomic techniques (NGTs)” that do not introduce foreign DNA, while maintaining the strict risk assessment for complex modifications. We also summarize the social, ethical, and public perception aspects of transgene-free gene-edited crops compared with traditional GMOs. Finally, we highlight the emerging role of AI in developing precise transgene-free gene-edited crops and the contributions these crops make to global food security. Collectively, this evidence supports the growing role of transgene-free gene-edited crops in scientific developments and real-world agricultural deployment, with remaining bottlenecks in delivery for recalcitrant crops, scalable and universal regulation, detection and traceability of the Cas footprints, and equitable access.

## 1 Introduction

Global food security is under increasing pressure in the 21^st^ century due to the convergence of demographic expansion, climate change, environmental degradation, and socioeconomic instability. The global population is projected to reach approximately 10 billion by 2050, which calls for a substantial increase in food production despite the limited agricultural land available (Wichelns, 2015). At the same time, climate change is intensifying abiotic stresses, including drought, heat, floods, and soil salinity, all of which are negatively impacting crop yields and productivity (Rathod and Verma, 2023). These stresses are particularly severe in arid and semi-arid regions where agriculture already operates near environmental limits. In parallel, global agriculture and food systems face increasing biotic pressures. For example, insect pests, plant pathogens, and weeds continue to evolve and spread, causing an estimated 20-40% loss in global crop production annually (Junaid and Gokce, 2024). The increased globalization of trade and climate-driven expansion of pests further increase the risks to food security and environmental protection. Meanwhile, chemical pesticide-based control of pests and yield enhancement has raised concerns about environmental sustainability, biodiversity loss, and human health impacts (Özkara et al., 2016).

Food security is also inseparable from nutritional security, as micronutrient deficiencies affect more than two billion people worldwide, underscoring the need not only for higher yields but also for improved nutritional quality of staple crops (Gautam et al., 2025). These challenges are further compounded by socioeconomic and political factors, including global conflicts, market volatility, and unequal access to technology, which affect smallholder farmers in developing countries (Borah et al., 2024). Addressing global food security, therefore, requires innovative, scalable, sustainable, and technologically advanced crop improvement strategies that deliver resilient, high-quality food under increasingly unpredictable conditions. Conventional breeding has contributed significantly to agricultural production for millennia and remains essential for crop improvement. However, its capacity to address the emerging challenges of food security and sustainable agriculture is limited. Conventional breeding depends on existing genetic variation and repeated cycles of crossing and selection, which are inherently time-consuming and constrained by reproductive barriers, long generation times, and linkage drag (Bharadwaj, 2018). These limitations become more pronounced in perennial crops, vegetatively propagated species, and polyploidy genomes, where breeding cycles may span decades. Moreover, biotic and abiotic stresses are often complex traits controlled by multiple genes, making them difficult to improve through conventional breeding.

The cost and duration of regulatory approval for transgenic crops are substantial, often exceeding a decade and requiring an investment of USD 100 million per trait, thereby effectively excluding smallholder farmers, public sector programs, and minor crops from commercialization pipelines (McDougal, 2011). For example, most of the transgenic crops, including Bt cotton, Bt corn, and herbicide-tolerant crops, are commercialized by multinational agricultural companies (ISAAA, 2017; Klümper and Qaim, 2014). Public perception, mandatory labeling, varying regulatory frameworks, and political opposition have further limited the adoption of transgenic crops, particularly in the EU, Asia, and parts of Africa, creating barriers to international trade and scientific innovations (Dessie and Zegeye, 2024). These constraints have driven the search for alternative, precise approaches to introduce and refine genetic modifications in crops, while reducing time, cost, and regulatory burdens, and enhancing public acceptance.

Gene editing technologies, particularly CRISPR-Cas, have fundamentally transformed plant biotechnology by enabling precise, efficient, and programmed modifications of endogenous genes to introduce desired traits (Ebrahimi and Hashemi, 2024; Zhu et al., 2020). CRISPR-Cas nucleases such as Cas9 and Cas12 are guided by small gRNAs to the specific genomic loci, where they introduce precise double-strand breaks (DSBs) that are repaired by endogenous cellular mechanisms, either by non-homologous end joining (NHEJ) or homologous directed repair (HDR) (Sultan et al., 2022). Endogenous repair of DSBs can be manipulated to introduce desired modifications. Unlike transgenic approaches, CRISPR-Cas systems can modify native alleles without introducing foreign coding sequences such as marker genes. CRISPR has transformed the genomic landscape of crops, with rapid developments in this field and a broad range of applications in plants, including disease resistance (Borrelli et al., 2018), abiotic stress tolerance (Zafar et al., 2020), nutritional improvement (Kumar et al., 2022; Liu et al., 2021), and altered plant architecture (Fernandes et al., 2025). A key advancement in this technology is the development of transgene-free gene-editing, defined as the generation of edited plants that do not stably integrate exogenous DNA into the final product. Transgene-free gene-editing outcomes can be achieved by segregating CRISPR cassettes after stable transformation, transient expression of CRISPR constructs, or direct delivery of CRISPR RNPs (Rocha et al., 2025). Among these approaches, direct delivery of CRISPR RNPs is particularly important because it limits the duration of nuclease activity and reduces the risks of random integration of foreign DNA into the host genome and off-target effects. Several crops have been modified using CRISPR RNPs to demonstrate the practical potential of this approach to generate transgene-free gene-edited crops; however, this approach makes the selection of modified plants a challenge (Ramakrishnan et al., 2025). Despite rapid progress, the literature on transgene-free gene-editing remains fragmented across molecular biology, crop science, regulatory classification, policy and governance, and socioeconomic disciplines. While several reviews address CRISPR-Cas developments and their applications in plant sciences (Chen et al., 2024; Sampath et al., 2023), a focused and comprehensive review of transgene-free gene-edited crops in the context of global food security, their regulatory classifications and frameworks, challenges, and the emerging role of AI in this field, is still lacking. The objectives of this review are fourfold. First, we focus on the conceptual, technical, and regulatory foundations of transgene-free gene-editing, including definitions, molecular mechanisms, regulatory classification, and delivery strategies that enable transgene-free outcomes. Secondly, we clarify the different approaches, including RNPs, transient expression of CRISPR cargoes, HI-edit, and transgene-killer technology, and summarize case studies on generating transgene-free gene-edited crops. Third, we examine the global regulatory landscape for gene-edited and transgene-free gene-edited crops, with a special focus on SDN-1 and SDN-2 transgene-free genome modifications. Finally, we summarize the social, ethical, and public aspects of transgene-free gene-edited crops; the role of AI integration with CRISPR in developing them; and their potential contribution to global food security, emphasizing sustainability, equity, and international trade.

## 2 Transgenic, cisgenic and transgene-free gene-edited crops: conceptual distinctions

Transgene-free gene-editing in plants refers to gene-edited plants that contain targeted genomic modifications but lack stably integrated foreign DNA in the genome, and the final product (Ahmad et al., 2023; Bhattacharjee et al., 2023). To appreciate the significance of a transgene-free gene-editing approach, it is essential to distinguish it from related breeding approaches, particularly transgenic and cisgenic technologies. From the GM plant’s perspective, crops involve the stable integration of one or more genes derived from the non-crossable species, typically using *Agrobacterium*-mediated transformation or particle bombardment. These genes remain permanently integrated and are inherited across generations, triggering a regulatory framework designed for genetically modified organisms (GMOs) (Altpeter et al., 2005; Gelvin, 2003). In contrast, cisgenic crops contain genes derived from the same species or from sexually compatible species, introduced using the same genetic engineering and transformation methods (Telem et al., 2013). While cisgenic avoids interspecies gene transfer, it still involves the stable integration of recombinant DNA (marker gene) into the host genome and therefore often falls under GMO regulation in many jurisdictions. From regulatory and social acceptance perspectives, cisgenic crops have not been shown to be different from transgenic crops, despite their conceptual relatedness to conventionally bred crops (van Hove and Gillund, 2017). Gene-edited crops differ fundamentally from both transgenic and cisgenic crops, and they sometimes do not retain any form of foreign DNA in the final product. In addition, the genetic change is limited to a targeted modification of the plant’s native genome. This distinction is not merely theoretical; it reflects a shift from random integration of foreign genes to precise genetic modification or optimization, aligning gene editing more closely with traditional breeding programs while leveraging molecular precision (Chen and Gao, 2020). A conceptual comparison of transgenic, cisgenic, gene-editing, and transgene-free gene-editing methods is summarized in **Table 1**.

**Table 1:** Comparison of gene editing, transgene-free gene-edited, transgenic, and cisgenic crops.

Features	Gene editing	Transgene-free gene-editing	Transgenic	Cisgenic
Methods	CRISPR/Cas, TALENs, and ZFNs are used for precise editing of DNA	CRISPR reagents are delivered as RNPs or transient DNA, with no integration into the genome	Agrobacterium or gene gun-mediated gene insertion often involves the permanent insertion of a transgene	Same as transgenic, but gene from the same/compatible species
Precision	Very high	Very high	Moderate (random insertion)	Moderate (random insertion)
Foreign gene	May or may not be present	No foreign DNA in the final product	Yes, from unrelated species	Yes, from the same or related species
Outcomes	Targeted mutations or allele changes	Natural-like mutations	Novel traits introduced	Existing traits are enhanced
Regulations	Country dependent	Often exempt or relaxed regulation	Strict GMO regulations	Usually regulated as GMOs
Developmental and regulatory cost	Low to moderate	Low	High	High
Public acceptance	Moderate	High	Low in some regions	Moderate
Concerns	Off-targets, ethics	Traceability, off targets	Potential environmental and health risks (no practical evidence)	Like transgenic, but with fewer ethical concerns
Biosafety and regulations	Risks are determined by the final product	Comparable to conventional breeding outcomes; often subject to proportionate regulation, but still requires case-by-case assessment	Strict regulatory oversight due to the stable insertion of foreign DNA	Holds an intermediate position biologically but is often regulated similarly to transgenic crops
Key insight	Precision breeding technology; risk assessment depends on the genetic modifications introduced	Precise and free of exogenous DNA, low risk, but with traceability challenges	Enables the introduction of novel traits, strict regulatory measures, and public concerns due to the presence of foreign DNA	The process uses genes from compatible species, but the final outcomes are often regulated similarly to transgenic crops
Reference	(Hsu et al., 2014)	(Gu et al., 2021)	(Turnbull et al., 2021)	(Holme et al., 2013)

## 3 Site-directed nucleases (SDNs) classification: SDN-1, SDN-2, and SDN-3

### 3.1 SDN-1

**Figure 1:**
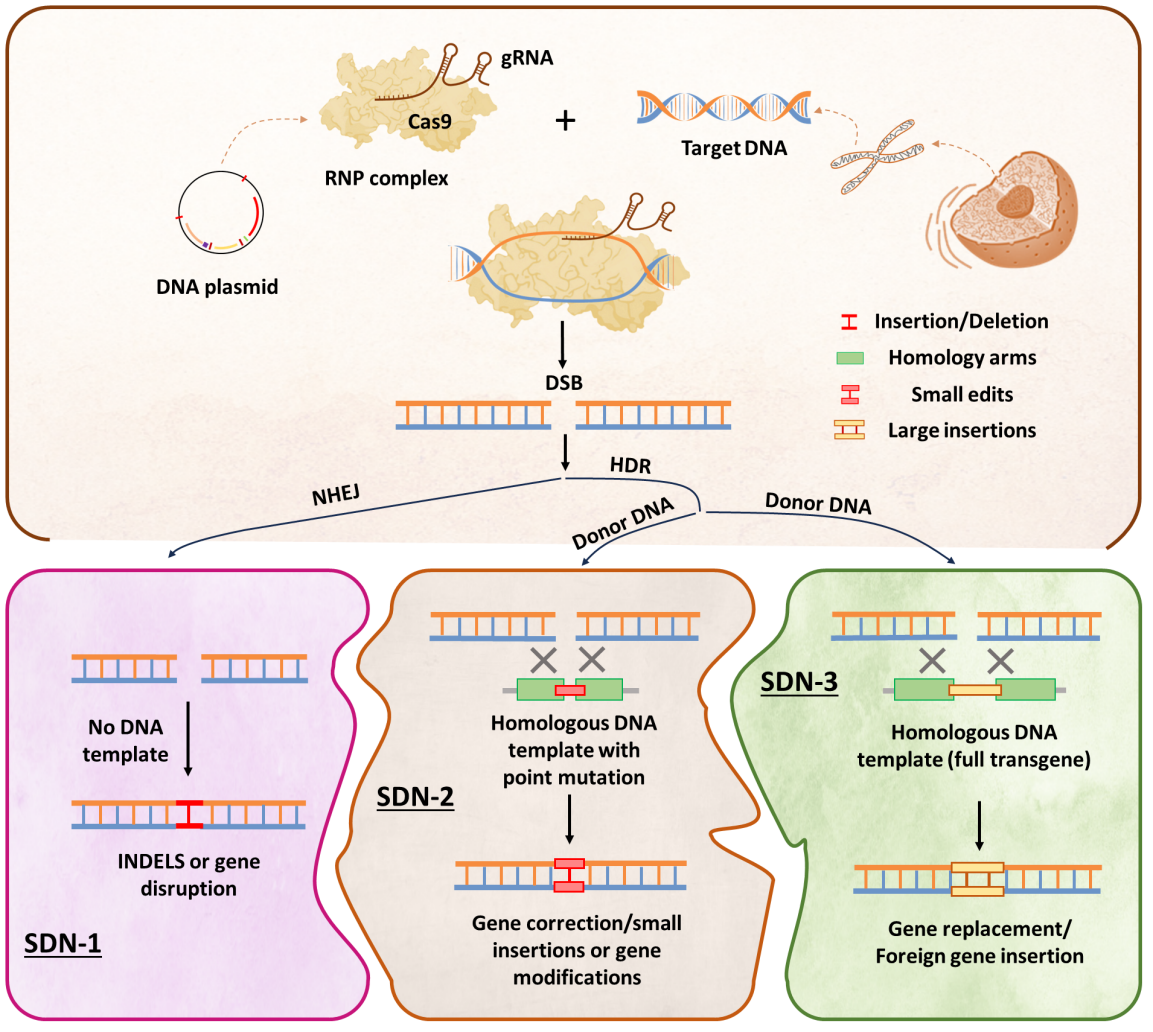
Diagrammatic representation of CRISPR-Cas9 genome editing and repair pathways. A plasmid DNA encodes a ribonucleoprotein (RNP) complex, which is composed of Cas9 and a guide RNA (gRNA), that targets double-stranded DNA to induce double-strand breaks (DSBs). These DSBs are subsequently repaired through two primary cellular pathways: non-homologous end joining (NHEJ) and homology-directed repair (HDR). NHEJ is an error-prone repair mechanism that can result in small insertions or deletions (indels), leading to gene disruption or gene knockout. It does not require the insertion of any DNA template, an outcome referred to as site-directed nuclease-1 (SDN-1). In contrast, HDR requires a homologous DNA template and is categorized into two further outcomes: SDN-2 and SDN-3. SDN-2 enables the introduction of a point mutation using a short homologous template, whereas the SDN-3 approach results in the insertion of an entire gene into the target locus.

SDN-1 modifications result from repair of the targeted DSB via the NHEJ repair pathway (**Figure 1**) and require no external or foreign DNA template. This repair commonly results in indels at the target site that disrupt gene functions, making it particularly relevant for creating knockout mutations to improve disease resistance or alter plant architecture.

### 3.2 SDN-2

Unlike SDN-1, SDN-2 modifications introduce precise nucleotide changes using a short homologous repair template that matches the target region but contains one or a few nucleotide mutations. These modifications generate specific allelic variants without introducing new genes. Although SDN-2 modifications are conceptually similar to natural point mutations, their reliance on a repair template raises regulatory concerns, particularly when the template is delivered as DNA. Nevertheless, emerging approaches using RNPs and transient expression suggest that SDN-2 modifications can also be achieved in a transgene-free manner. SDN-2 modifications enable the precise introduction of desirable traits, such as enhanced drought resistance in maize (Shi et al., 2017) or herbicide tolerance and disease resistance in rice (Sun et al., 2016; Wei et al., 2021).

### 3.3 SDN-3

SDN-3 modifications involve the insertion of large DNA fragments, such as entire gene(s) or promoters, at the target site using a homologous repair template. Even when precisely targeted, SDN-3 outcomes typically result in stable integration of foreign DNA into the host genome and are therefore generally excluded from the definition of transgene-free gene-editing. Therefore, SDN-3 edits are strictly regulated as with transgenic GM crops. A comparison of the SDN-1, SDN-2, and SDN-3 modifications is summarized in **Table 2**.

**Table 2:** A comparison of SDN-1, SDN-2, and SDN-3 modifications.

Feature	SDN-1	SDN-2	SDN-3
Type of genome modification	Small insertion or deletion (Indels)	Precise, small sequence modifications	Large or novel DNA insertions
DNA template used	NO	Yes (short repair template)	Yes (large repair template)
Repair mechanism	NHEJ	HDR	HDR
Precision of edit	Low to moderate	High	High
Foreign DNA integration	No	No (typically)	Yes
Typical edit size	1-few bps	Few bps	Large gene or multiple genes
Resulting organism	Mimics natural mutation	Mimics natural mutations	GMOs (transgenic or cisgenic)
Common applications	Gene knockout	Gene corrections or allele replacement	Trait introduction
Regulatory trend	Often exempt from GMO regulation, greater regulatory and commercial advantages	Exempt or case-by-case regulation represents a regulatory gray zone, with inconsistencies among jurisdictions	Regulated as a GMO, faces the highest regulatory burden, public scrutiny, and commercialization costs
Biosafety consideration	Comparable to naturally occurring or conventionally induced mutations, with a low risk of potential off-target mutations, and confirmation of no exogenous DNA	Similar concerns as SDN-1, but with additional consideration of repair-template-associated modifications, case-by-case assessment of intended edits	Comprehensive biosafety assessments are required due to the insertion of large DNA fragments that may alter gene expression, metabolic pathways, or ecological interactions
Example	Gene knockout (non-browning mushrooms)	Changing one bp to improve enzyme efficiency (*ALS* gene modification in rice)	Adding the Bt gene or the herbicide tolerance gene

## 4 CRISPR cargoes to achieve transgene-free gene-edited crops

Transgene-free gene-editing can be achieved through multiple experimental approaches, each with distinct technical and practical implications. One common approach involves stable transformation with an expression plasmid DNA, followed by genetic segregation, in which CRISPR constructs are introduced into the plant genome to make edits and then removed through sexual reproduction (He and Zhao, 2020). While effective for annual, sexually reproducing crops, this approach is less suitable for vegetatively propagated species, perennials, or elite cultivars, where backcrossing is undesirable or impractical. An alternative strategy is the transient expression of CRISPR components, in which editing machinery is expressed temporarily without stable integration into the host genome (Yarra and Krysan, 2025). Transient expression can be achieved using non-integrating DNA vectors, RNA delivery, or viral replicons. Edited plants are then screened to identify individuals lacking the CRISPR cassette. Although this approach avoids long-term transgene presence, it still requires careful molecular screening to exclude unintended integration events. The most direct route to transgene-free gene-editing is DNA-free delivery of CRISPR RNPs (Ramakrishnan et al., 2025). In this approach, a purified Cas protein is preassembled with gRNA and delivered directly into plant cells, where it performs gene editing before being rapidly degraded. RNPs-based editing minimizes the duration of nuclease activity, reduces off-target risks, and eliminates the possibility of foreign DNA integration. Consequently, CRISPR RNP delivery is increasingly considered as the standard method for generating transgene-free gene-edited crops, despite ongoing challenges related to delivery efficiency, screening of edited plants, and plant regeneration (Cai et al., 2025; Gu et al., 2021).

## 5 CRISPR systems enabling transgene-free gene-editing in crops

### 5.1 CRISPR-Cas9: the most widely used system for transgene-free gene-editing

Cas9 is particularly well-suited for transgene-free gene-editing because it can be delivered as a purified Cas9-gRNA RNP complex. RNP delivery yields transient nuclease activity, thereby reducing off-target effects and eliminating the risk of unintended DNA integration (Guo et al., 2023). Cas9 RNP-mediated editing has been successfully demonstrated in crops, including rice, wheat, maize, lettuce, and potato, using protoplast transfection or particle bombardment (Zhang Y et al., 2021). However, Cas9 has limitations in transgene-free gene-editing applications. The strict 5’-NGG-3’ PAM requirement restricts target site availability in some genomic regions, particularly in AT-rich plant genomes. In addition, DSB repair via NHEJ can produce heterogeneous outcomes, including large deletions or chromosomal rearrangements, necessitating rigorous molecular characterization of edited plants. These limitations have driven the development of alternative CRISPR-Cas systems with distinct biochemical properties. CRISPR-Cas9 has been extensively used for transgene-free gene-editing in plants, including rice (Aliaga-Franco et al., 2019), wheat (Zhang et al., 2016), tomato (Veillet et al., 2019), maize (Svitashev et al., 2016; Zhang et al., 2020), and carrot (Yarra and Krysan, 2025).

### 5.2 CRISPR-Cas12 systems: expanding the CRISPR toolkit for transgene-free gene-editing

Cas12a-mediated staggered cuts may improve certain editing outcomes, particularly for targeted deletions or insertions (Ji et al., 2026). Moreover, the smaller size of Cas12 variants makes them better suited for RNP delivery and emerging non-DNA delivery strategies. Cas12 has been successfully applied to transgene-free gene-editing in crops such as rice and maize, achieving efficient SDN-1 modifications comparable to Cas9 (Dong et al., 2021; Zhang et al., 2022). As engineered and PAM-flexible Cas variants continue to be developed, Cas12 systems are expected to play an increasingly important role in transgene-free plant gene editing, particularly in species where Cas9 target availability is limited. CRISPR-Cas12 has been used for transgene-free gene-editing in plants such as citrus (Su et al., 2024; Zhang et al., 2022), *Nicotiana benthamiana* (Banakar et al., 2022; Blumberg et al., 2026), and soybean (Banakar et al., 2022).

### 5.3 Cas13: RNA targeting through CRISPR-Cas

Cas13 is an RNA-targeting nuclease, so the concept of transgene-free gene-editing in the conventional DNA-editing sense does not strictly apply to this RNA-editing Cas. RNA-targeting CRISPR systems, particularly Cas13, provide a complementary approach to DNA editing by enabling programmable RNA cleavage without altering genomic DNA. Cas13 can be introduced into plant cells via *Agrobacterium* transformation or the biolistic method, using a DNA construct encoding Cas13 and crRNA. Alternatively, transient delivery of Cas13 mRNA or preassembled ribonucleoprotein (RNP) complexes to protoplasts may enable RNA targeting without stable genomic integration. Once inside the cell, Cas13 binds to its crRNA, recognizes the complementary target RNA, and cleaves it. From a transgene-free gene-editing perspective, Cas13 systems are inherently attractive because they do not introduce heritable genomic changes, thereby eliminating concerns about permanent off-target mutations or genomic instability. In plants, Cas13 has been explored primarily for antiviral applications (Kavuri et al., 2022), where it can target RNA viruses responsible for significant yield losses. However, because Cas13-mediated effects are not inherited, continuous expression or repeated delivery is required for durable phenotypic outcomes. Consequently, Cas13 systems are best viewed as complementary tools for disease management and functional genomics rather than a replacement for transgene-free DNA-editing approaches in crop breeding.

### 5.4 Base editing: precision without introducing a DSB

In plants, base editors have been used to generate agriculturally relevant traits, including herbicide resistance (Peng et al., 2025; Wei et al., 2023; Zheng et al., 2026) and yield-associated alleles (Hao et al., 2019; Hua et al., 2018). Conceptually, base editing aligns closely with SDN-2 modifications because it introduces precise sequence changes that often mimic naturally occurring variants. Although base editing has been used for base substitution in crops to introduce desired traits (Cheng et al., 2025; Das et al., 2026), technically, transgene-free base editing is technically challenging because base editors are large fusion proteins that are difficult to deliver as RNPs.

### 5.5 Prime editing

Prime editing combines a Cas9 nickase (nCas9) with a reverse transcriptase and a prime editing gRNA (pegRNA) to enable precise insertions, deletions, and base substitutions without inducing double-strand breaks or requiring a donor DNA template. This technology is substantially expanding the range of programmable gene modifications (Anzalone et al., 2019). The prime editing construct is designed by combining the CRISPR-Cas9 nickase (nCas9) with the reverse transcriptase domain, thereby enabling accurate DNA modification without inducing double-strand breaks. It uses a prime editing guide RNA (pegRNA) that contains the spacer sequence for target recognition, an extended template sequence encoding the desired modification, and a primer binding site. In some designs, an additional nicking sgRNA is added to stimulate repair of the modified strand and increase editing efficiency. Prime editing has been demonstrated in crop species, including rice and wheat (Lin et al., 2020) and tomato (Van Vu et al., 2026). However, it is less efficient than conventional CRISPR-Cas9 editing. From a transgene-free gene-editing perspective, prime editing offers considerable promise but also significant challenges. The large size and complexity of the prime editing construct necessitate the DNA-based delivery in most plant systems (Lee et al., 2025). If scalable DNA-free prime editing becomes practical in plants, it would represent a breakthrough in precision breeding and regulation-friendly crop improvement (Chavhan et al., 2025). We can expect that advanced prime editing systems with optimized delivery and expression may overcome these barriers in the future.

### 5.6 Multiplex gene editing: the future of gene editing technology

Multiplex gene editing is achieved by delivering multiple gRNAs in a single editing event across crops, such as rice (Guan et al., 2026), wheat (Abdallah et al., 2025; Sánchez-León et al., 2024), tomato (Berman et al., 2025; Sethi, 2024), soybean (Li N et al., 2025; Liu T et al., 2025), and Brassica (Sahab et al., 2024). In transgene-free gene-editing, this is typically achieved via RNP delivery or transient expression systems; however, efficiency often declines as the number of targets increases. Nevertheless, a successful example demonstrates that multiplex gene editing can be achieved without foreign DNA integration (Weiss et al., 2026). The ability to engineer multiple traits within a single generation represents a paradigm shift in plant breeding. As delivery technologies and gRNA design algorithms continue to improve, multiplex transgene-free gene-editing is poised to become a powerful strategy for developing transgene-free gene-edited crops with desired traits, a capability that is particularly important for food security.

## 6 Methods for producing transgene-free gene-edited crops

### 6.1 RNPs

The use of CRISPR cargoes, such as RNP complexes, is among the most promising transgene-free methods for generating CRISPR crops. The RNP complex consists of the Cas protein, which acts as a molecular scissor, and an *in vitro*-transcribed gRNA that directs the Cas protein to the target site in the genome. By directly inserting RNPs into plant cells, researchers can achieve specific genetic alterations without introducing foreign DNA into the plant’s genome, yielding a truly transgene-free plant (Lee et al., 2020; Zhang et al., 2025). These RNP complexes are typically delivered into plant protoplasts by electroporation or PEG-mediated transformation. Electroporation transiently permeabilizes the cell membrane, enabling RNP entry, while PEG-mediated transformation promotes RNP association and uptake through membrane fusion. Following editing, the protoplasts are regenerated into complete plants using highly sophisticated tissue culture techniques that induce cell division and the development of plantlets (Subburaj and Agapito-Tenfen, 2023).

Using RNPs as CRISPR cargoes minimizes unintended genomic alterations, lowers regulatory barriers, and improves the social acceptance of the resulting products (Seijas et al., 2025). Because RNPs persist in cells for only a short time, the risk of off-target editing is significantly reduced, thereby increasing the precision and efficiency of genome editing. Notably, researchers have successfully generated transgene-free rice (Li et al., 2020; Loo et al., 2025) and tomato (Nekrasov et al., 2017) plants exhibiting enhanced resistance to bacterial blight and powdery mildew, respectively. Despite these advantages, RNP-based editing faces several challenges. The efficiency of tissue culture protocols for regenerating edited plants varies among species, and some crops, such as cereals and woody plants, have recalcitrant protoplast systems that hinder RNP delivery. Additionally, purifying Cas proteins for RNP preparation remains both costly and technically demanding. Nevertheless, RNP-mediated editing continues to gain traction as a preferred approach, enabling precise, transgene-free modifications while minimizing regulatory complexities.

### 6.2 Transgene killer systems

Another innovative approach to developing transgene-free CRISPR-edited crops is the transgene-killer system, which uses transient expression of CRISPR-Cas components followed by degradation of any foreign DNA introduced during editing (Bhattacharjee et al., 2023; Saniya et al., 2025; Zhu et al., 2025). This method balances editing efficiency with the production of a non-transgenic final product, particularly in crops with complex regeneration systems. The process typically begins with delivering Cas9 and gRNA into plant cells *via Agrobacterium*-mediated plasmid transformation. Notably, this strategy is recognized as an effective means of achieving transgene-free genome editing in agricultural biotechnology. Under this approach, CRISPR components are expressed transiently, and subsequent generations of edited plants are screened to identify progeny free of foreign DNA. Transgene elimination occurs through segregation, in accordance with Mendelian inheritance patterns, or through excision-based methods that actively remove foreign DNA from the genome (Bhattacharjee et al., 2023).

The transgene killer cassette (TKC) system is designed to automatically eliminate transgene-containing gametes in embryos by coupling CRISPR editing machinery with suicide genes driven by tissue-specific promoters. Typically, the T-DNA construct harbors (i) the CRISPR-Cas9 editing module and (ii) one or more cytotoxic genes, such as *barnase* or other embryo-or pollen-lethal genes, placed under the control of gametophyte-or embryo-specific promoters. During transformation, gene editing occurs in somatic tissues; however, when the transgene is transmitted to reproductive tissues, the killer gene becomes active, causing selective abortion of transgene-carrying pollens, ovules, or embryos. As a result, only progeny that have inherited the desired gene edits but lack the T-DNA cassette survive. This built-in genetic counter-selection system markedly accelerates the recovery of transgene-free gene-edited plants within a single generation and minimizes the need for extensive segregation screening (He et al., 2018; Yubing et al., 2019).

Transgene killer technology offers several advantages over other methods. It is highly efficient across a wide range of plants, including major crops such as rice and maize. It does not require complex tissue culture or protoplast regeneration, both of which are technically demanding and species-specific. For example, this technology was successfully used to edit tomato plants to enhance disease resistance by transiently transfecting CRISPR components, followed by the screening of successive generations to segregate the transgenic elements (He et al., 2018; Yubing et al., 2019).

**Figure 2:**
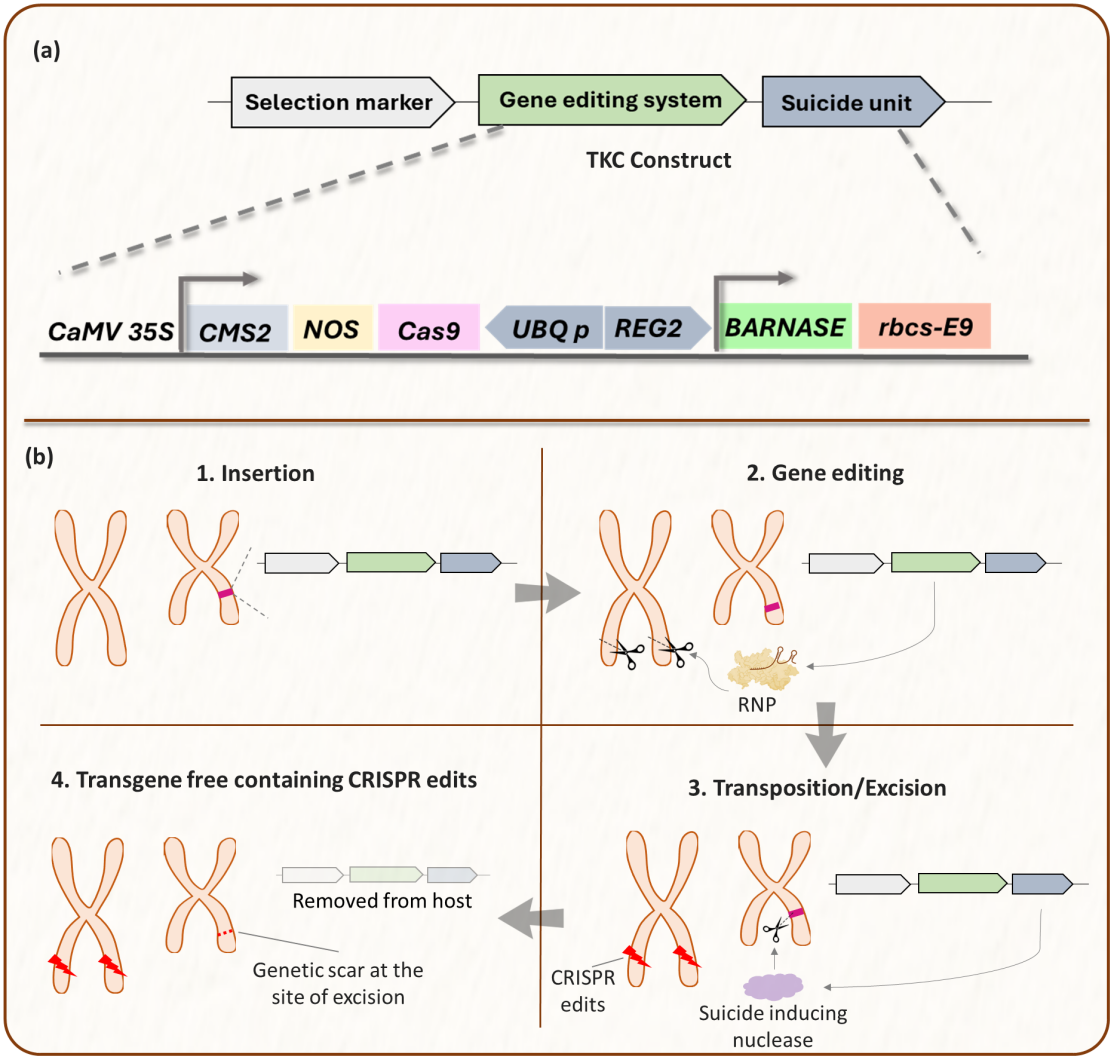
Diagrammatic illustration of transgene deletion using the transgene killer CRISPR (TKC) system. **(A)** The TKC construct contains a selection marker, a CRISPR gene-editing module, and a suicide gene. The suicide components REG2:*BARNASE* and 35S:*CMS2* induce embryo and pollen lethality, respectively, thereby preventing the formation of transgenic seeds in T_0_ plants. **(B)** When introduced into the plant *via Agrobacterium*-mediated transformation, the TKC construct enables genome editing via a Cas9/gRNA complex. Subsequently, the integrated transgenes flanked by the T-DNA borders are excised from the host genome, leaving a small genetic “scar” at the excision site.

Despite these significant advantages, the transgene killer system has limitations. Segregation or removal of foreign DNA is often time-consuming, sometimes requiring several generations to identify and propagate transgene-free plants. This challenge is particularly acute for crops with long reproductive cycles or low seed production. Furthermore, avoiding unintended genetic effects from the transiently expressed components requires tight control of their expression. Nevertheless, the transgene-killer system appears highly promising for producing transgene-free gene-edited crops, as shown in **Figure 2**. This approach combines the efficiency of *Agrobacterium*-mediated transformation with the subsequent removal of transgenic elements, making it a robust strategy for developing crops that are both regulatory-compliant and consumer-preferred.

### 6.3 Crossing to segregate the transgene from the edited plants

The crossing strategy is a classic and efficient method for generating transgene-free CRISPR crops by combining traditional plant breeding with modern genome-editing technologies. This approach begins by generating gene-edited plants using standard transformation techniques, such as *Agrobacterium*-mediated or biolistic methods. These methods incorporate plasmids carrying the Cas cassette and gRNA into the plant genome to induce the desired modifications. After successful editing, the modified plants that temporarily carry the transgenes are crossed with wild-type (non-edited) plants (Abdallah et al., 2024; Kocsisova and Coneva, 2023). This cross facilitates genetic recombination, allowing the transgenic components (Cas cassette and gRNA) to segregate independently of the desired edits in the offspring. Subsequent generations are screened to identify progeny that retain the specific genomic edits but are completely free of the Cas gene and gRNA (Bhattacharjee et al., 2023).

The major advantage of this approach is its broad applicability across a variety of crop plants, including those resistant to direct transformation methods. This technique has already been successfully used in rice and wheat breeding programs to develop new varieties with increased resistance to several diseases and improved grain traits. Because the crossing approach relies entirely on natural genetic recombination, no additional molecular manipulations are required to eliminate the transgenes, simplifying the regulatory approval process (Gu et al., 2021). Despite these advantages, the crossing strategy has notable drawbacks. It is time-consuming and requires multiple generations to produce transgene-free offspring. This limitation is particularly significant for plants with long reproductive cycles, such as fruit trees and certain perennial crops. The process also depends on the availability of compatible wild-type lines with appropriate characteristics for crossing, which is not always guaranteed. Nonetheless, the crossing strategy remains an effective means for generating transgene-free gene-edited crops, especially when alternative technologies are neither economical nor very efficient.

### 6.4 mRNA-based genome editing

**Figure 3:**
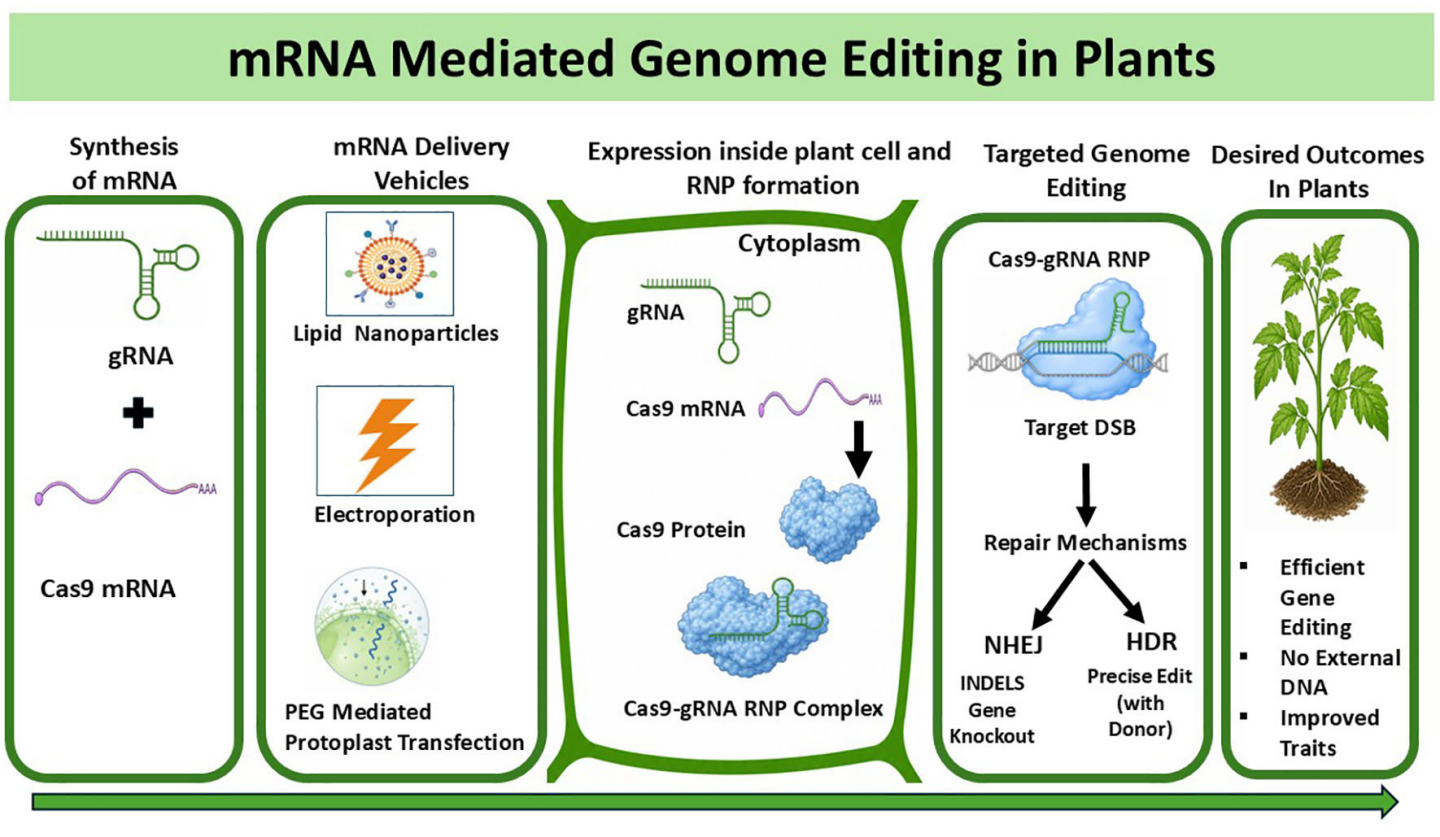
mRNA-mediated transgene-free gene-editing in plants. *In vitro*-transcribed mRNA encoding gRNA and Cas9 is delivered to plant cells by various methods. Inside the plant cell, Cas9 mRNA is translated into the Cas protein, which then combines with gRNA to form an active RNP complex that enables targeted gene editing.

**Figure 4:**
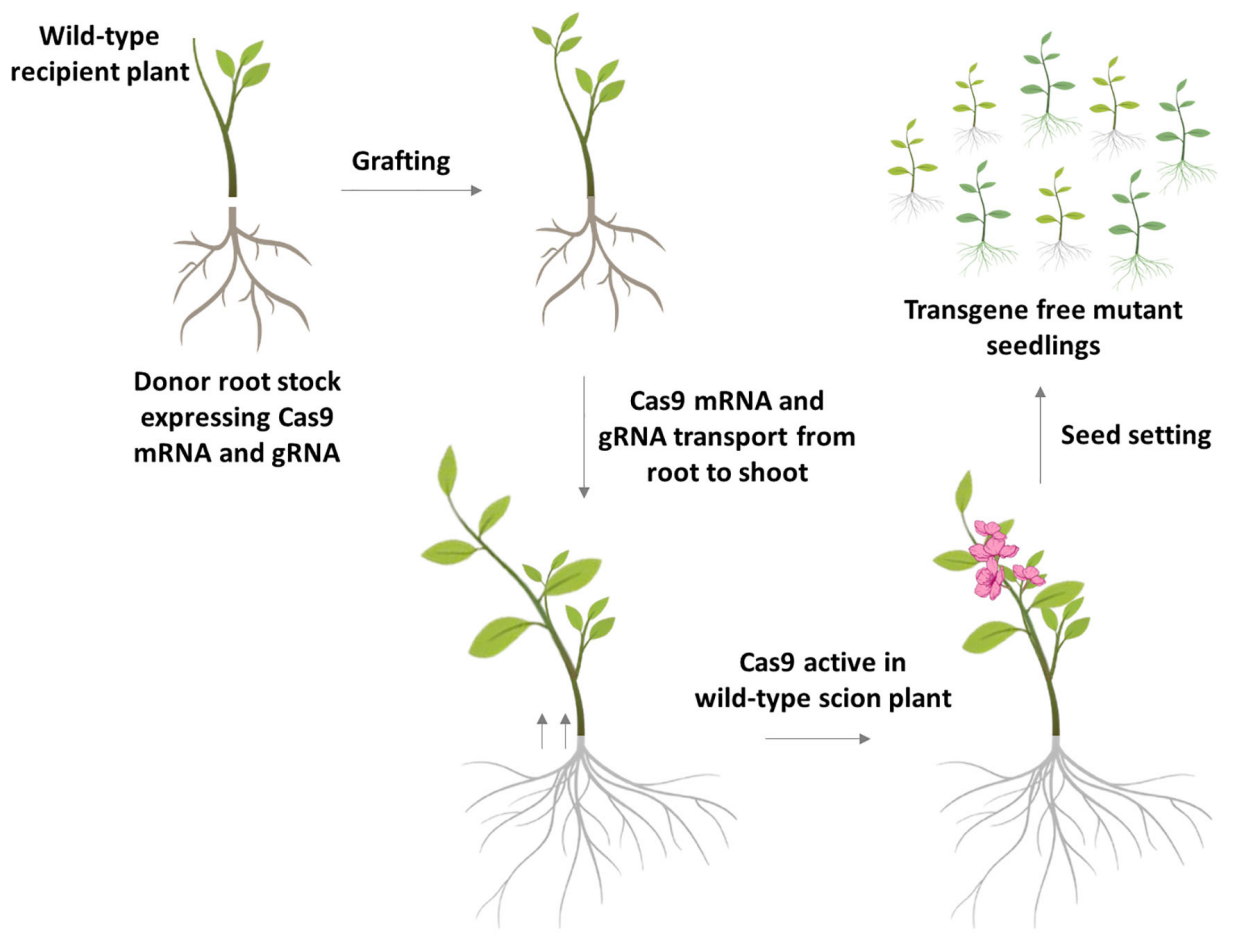
Grafting-based delivery of CRISPR mRNA for transgene-free genome editing. Cas9 mRNA and guide RNA (gRNA) are expressed in the rootstock. A wild-type scion is grafted onto the rootstock. CRISPR RNAs migrate into the scion’s reproductive tissues, where they mediate genome editing. Transgene-free, genome-edited plants are obtained in the next generation.

The mRNA-based approach is a viable technique for generating transgene-free gene-edited crops, leveraging the transient nature of messenger RNA (mRNA) to introduce CRISPR-Cas9 components into plant cells without inserting foreign DNA into the genome (Huang et al., 2023; Qiu et al., 2025). In this strategy, synthetic mRNA encoding the Cas9 nuclease is delivered into the plant cell along with a gRNA, and is subsequently translated to execute genome editing (Yang et al., 2023). Its principal strength is that the mRNA naturally degrades after inducing the desired gene modification, leaving no transgenic remnants in the resulting plant, as illustrated in **Figures 3**, **4**. This approach has been successfully implemented in plants such as rice and maize, where direct microinjection or nanoparticle-mediated delivery of mRNA has achieved accurate genome edits without stable transformation (Gong et al., 2021). Furthermore, compared with plasmid-delivery systems, mRNA-delivery-based genome editing is less likely to generate off-target effects and presents fewer controversial regulatory challenges, making it a highly promising means for commercial crop enhancement.

### 6.5 Transient expression using viral vectors

Transient expression *via* viral vectors is another useful approach for generating transgene-free gene-edited crops, enabling temporary expression of CRISPR components without irreversible genetic modification (Bhattacharjee et al., 2023; Kocsisova and Coneva, 2023). In this process, plant viruses genetically modified to carry the CRISPR-Cas9 system infect plant cells. Inside the cells, the viruses transiently express the required genome-editing components, which are subsequently degraded naturally. Because the viral genome does not integrate into the plant’s DNA, the progeny of the edited plants is free from foreign genetic material. This strategy has been successfully used in plants such as tomato, *benthamiana*, and bamboo, where viral vectors such as Potato Virus X (PVX) and Bamboo Mosaic Virus (BMV) have facilitated highly efficient, accurate editing of target genes without stable transformation (Lee et al., 2024; Liu et al., 2026; Wu et al., 2025). This approach is particularly beneficial for species that are difficult to regenerate using protoplast-based procedures, providing a non-invasive and scalable route for accurate genome editing in crop plants.

### 6.6 Paraquat resistant 1-based positive screening (PARS)

A second efficient approach to producing transgene-free gene-edited crops is Paraquat Resistant 1 (PAR1)-based Positive Screening (PARS). This method exploits a mutation in the *PAR1* gene, which encodes an L-type amino acid transporter, to confer resistance to the herbicide paraquat. Because paraquat is lethal to all wild-type green plants, only those carrying the mutated gene can survive. In the PARS approach, CRISPR-Cas9 components are transiently introduced to induce targeted mutations in *PAR1*. After transformation, seeds are plated on paraquat-containing medium, allowing only successfully edited plants to survive the selection. PCR screening is then performed to detect plants devoid of the Cas9 transgene, ensuring that the final progeny is transgene-free. This approach greatly improves screening efficiency, increasing the recovery of transgene-free plants by up to 2.81-fold, and is applicable across several crop species (Kong et al., 2023). Mechanistically, the PARS strategy relies on CRISPR-Cas9-mediated disruption of the *PAR1* gene, which encodes an L-type amino acid transporter implicated in paraquat uptake and intracellular trafficking. In wild-type plants, paraquat enters cells and is transported to chloroplasts, where it accepts electrons from photosystem 1, generating excessive reactive oxygen species (ROS) that lead to lipid peroxidation, membrane damage, and rapid cell death. Targeted knockout of *PAR1* reduces paraquat transporter efficiency, limiting its accumulation in chloroplasts and thereby preventing ROS overproduction. During transient CRISPR delivery, edited cells acquire paraquat tolerance and survive on selective media, while non-edited cells die, enabling enrichment of transgene-free mutants after segregation of Cas9 components (Kong et al., 2023). Paraquat is uniquely suited for PAR1-based positive screening because it is extremely toxic to wild-type green plants, creating strong, clear selection pressure, in which only *PAR1*-edited mutants survive. Resistance results from a simple loss-of-function mutation in the PAR1 transporter gene, enabling efficient recovery of edited, transgene-free plants, without introducing foreign resistance genes. Although other herbicides could theoretically be used, most require specific point mutations, a transgene-based detoxification system, or show weaker selection stringency, making them less suitable than paraquat for this strategy.

### 6.7 Haploid induction editing technology (HI-edit)

**Figure 5:**
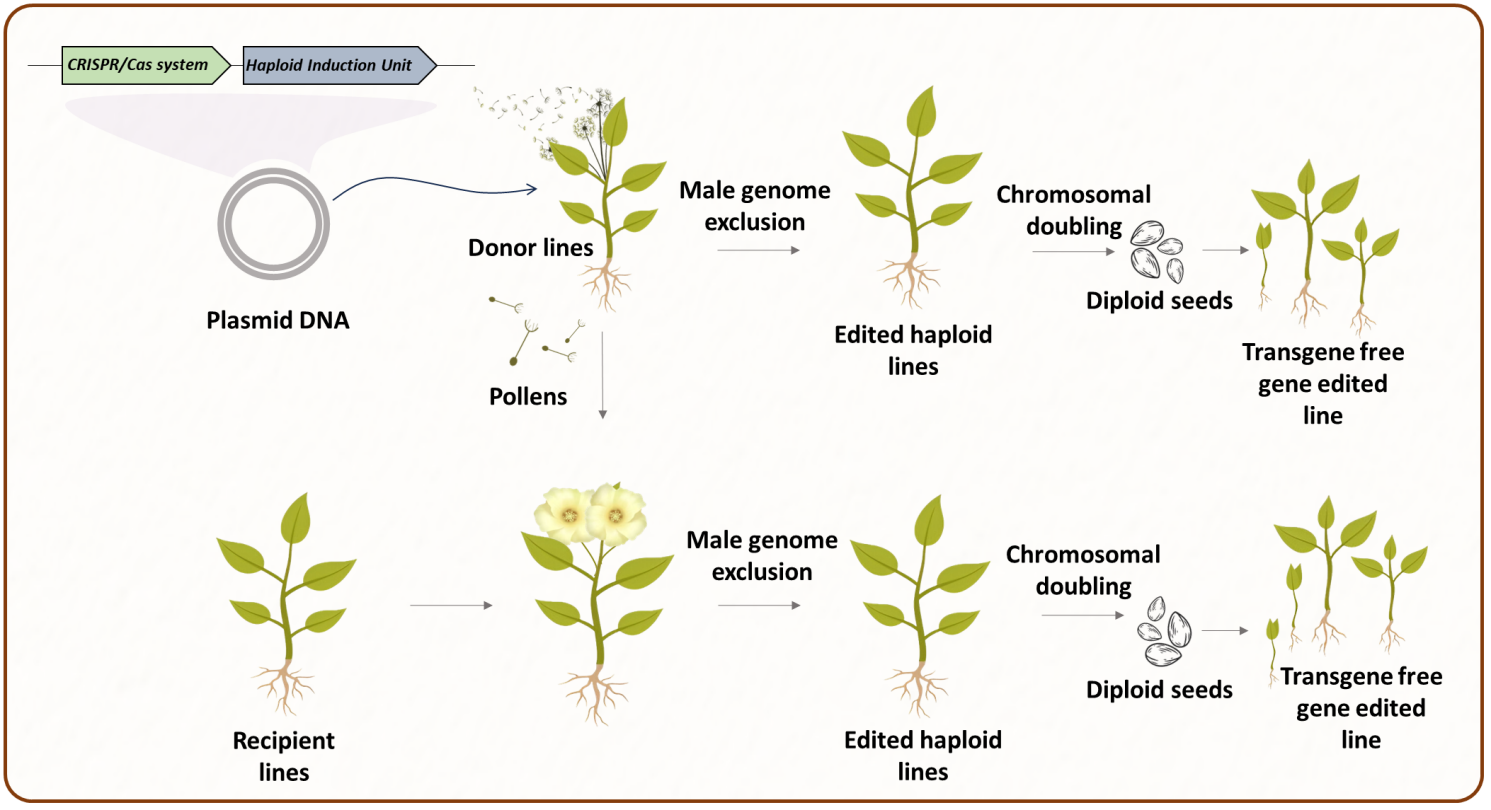
Coupling genome editing with haploid induction to generate transgene-free edited plants. Plasmid DNA carrying the CRISPR/Cas system and the haploid induction cassette is delivered into donor plants *via Agrobacterium*-mediated transformation. Donor lines expressing Cas9 and guide RNA (gRNA) then serve as pollen donors for recipient lines. Following fertilization, the male genome is selectively eliminated, yielding haploid lines that are both gene-edited and transgene-free. Subsequent chemical chromosome doubling produces diploid seeds that germinate into transgene-free plants carrying the desired mutation.

Haploid induction has emerged as a powerful strategy for generating transgene-free gene-edited crops by combining gene editing with double haploid technology, thereby accelerating breeding cycles and eliminating foreign DNA in a single generation (Kelliher et al., 2019). In this approach, CRISPR-Cas components are introduced into a haploid inducer line, often via stable transformation, targeting either a trait gene of interest or a haploid induction gene such as *MTL*/*ZmPLA1*/*NLD* in maize (Sheng et al., 2025). When this edited inducer line is crossed with an elite, non-transgenic recipient line, genome editing can occur in the zygote shortly after fertilization. Subsequently, selective elimination of the inducer genome yields haploid progeny that retain only the recipient’s parental chromosomes but harbor the desired CRISPR-induced mutation (He et al., 2022; Li H et al., 2025). Because the editing machinery is carried on the inducer genome, which is later lost during genome elimination, the resulting haploid plants are free of integrated transgenes. Chromosome doubling, either spontaneously or via colchicine treatment, produces fertile, homozygous double haploid lines in a single generation. This strategy, sometimes referred to as HI-edit or haploid inducer-mediated gene editing (IMGE), has been successfully demonstrated in crops such as maize (Sheng et al., 2025), wheat (Karmacharya et al., 2023), and cabbage (Li Y et al., 2025), significantly shortening breeding timelines compared to conventional segregation-based approaches. Importantly, haploid induction minimizes linkage drag and reduces the need for prolonged backcrossing, making it particularly attractive for complex traits and hybrid breeding programs. However, challenges remain, including optimizing editing efficiency during early zygotic development, expanding the use of induction systems beyond major cereals, and ensuring regulatory clarity regarding transient transgene exposure. Nonetheless, HI-based genome editing represents a transformative pathway towards rapid transgene-free gene-edited crop improvement (**Figure 5**).

## 7 Transgene-free gene-editing in crops: case studies

Transgene-free gene-editing has evolved from early demonstrations in model species such as *Arabidopsis* to a growing portfolio of commercial crops, including wheat, rice, maize, horticultural crops, and perennial fruit crops. This section summarizes practical examples of translational applications of transgene-free gene-editing in crops (**Table 3**).

**Table 3:** Transgene-free gene-editing studies in crops.

Crop	CRISPR system used	CRISPR reagent	Transgene-free gene-editing method	DSB repair pathway	Type of Modifications	Target gene/genes	Outcome	Reference
Rice (*Oryza sativa*)	CRISPR/Cas9	CRISPR RNPs	PEG-mediated protoplast transfection	NHEJ	SDN-1	*P450; DWD1*	DNA-free targeted mutagenesis	(Woo et al., 2015)
Rice (*Oryza sativa*)	CRISPR/nCas9- deaminase fusion	*Agrobacterium*	Segregation	Base editing	SDN-2	*OsALS/ALS*	Herbicide-tolerant rice	(Zhang R et al., 2021)
Rice (*Oryza sativa*)	CRISPR/Cas9	*Agrobacterium*	DSRED negative	NHEJ	SDN-1	*Os04g56950*	Transgene-free mutants	(Aliaga-Franco et al., 2019)
Rice (*Oryza sativa*)	CRISPR/nCas9-PE	*Agrobacterium*	Cas9 DNA- pollen-killer	NHEJ	SDN-1	*EUI1; PTC1*	Transgene-free mutants	(Yu et al., 2024)
Rice (*Oryza sativa*)	Prime Editor (PE3max)	*Agrobacterium*-mediated transient transformation	PE vector transient	Prime editing repair	SDN-2	*OsALS; OsEPSPS; OsXa5; OsCold1*	Transgene-free T0 plants	(Lu et al., 2025)
Rice (*Oryza sativa*)	CRISPR-Cas9	Cas9-gRNA construct	Transgene Killer Technology (TKC)	NHEJ	SDN-1	*LAZY1*	Transgene-free lazy1 mutants	(He et al., 2018)
Wheat (*Triticum aestivum*)	CRISPR/Cas9	Cas9 RNPs	Particle bombardment	NHEJ	SDN-1	*TaGW2; TaGASR7*	DNA-free wheat mutants	(Liang et al., 2017)
Wheat (*Triticum aestivum*)	CRISPR/Cas9	Viral expressed Cas9 and gRNA	Viral RNA delivery (BYSMV RNA virus)	NHEJ	SDN-1	*TaPDS/TaSDN1*	Heritable virus-free mutants	(Qiao et al., 2025)
Wheat (*Triticum aestivum*)	CRISPR/Cas9	Cas9 DNA/RNA and gRNA	Transient (DNA/RNA-based) expression	NHEJ	SDN-1	*TaGASR7; TaGW2*	Transgene-free homozygous mutants	(Zhang et al., 2016)
Wheat (*Triticum aestivum*)	CRISPR/Cas9	Cas9/gRNA construct	Segregation	NHEJ	SDN-1	*TaQsd1*	Transgene-free dormant mutants	(Abe et al., 2019)
Maize (*Zea mays*)	CRISPR/Cas9	CRISPR RNPs	Particle bombardment	NHEJ	SDN-1	*LIG; ALS2; MS26; MS45*	DNA-free maize mutants	(Svitashev et al., 2016)
Maize	CRISPR-Cas12a	CRISPR-RNPs	Particle bombardment	NEHJ	SDN-1	*Bx9; MIR604*	Heritable DNA-free mutants	(Dong et al., 2021)
Maize	CRISPR/Cas9	Cas9-gRNA construct	Segregation	NHEJ	SDN-1	*ZmGA20ox3*	Transgene-free semidwarf maize	(Zhang et al., 2020)
Maize	CRISPR/cas9	Cas9-gRNA construct	Segregation	NHEJ	SDN-1	*MS8/ZmMS8*	Transgene-free male sterility	(Chen et al., 2018b)
Tomato (*Solanum lycopersicum*)	CRISPR/Cas9	CRISPR-RNPs	Protoplast transfection	NHEJ	SDN-1	*SP; SP5G*	DNA-free tomato mutants	(Liu et al., 2022)
Tomato (*Solanum lycopersicum*)	CRISPR/Cas9	Virus expressed Cas9 and gRNA	RNA virus-based delivery	NEHJ	SDN-1	*SlPDS; SlDA1*	Virus-free homozygous mutants	(Liu Y et al., 2026)
Tomato (*Solanum lycopersicum*)	CRISPR-nCas9	Base editing construct	Segregation	Base editing	SDN-2	*SlALS/ALS1*	Chlorsulfuron-resistant tomato	(Veillet et al., 2019)
Tomato (*Solanum lycopersicum*)	CRISPR/Cas12a	Cas12a construct	Segregation	Base editing/NHEJ	SDN-1/SDN-2	*SlALS1; SlER; SlRBL2; SlRbohD*	Transgene-free T0 mutants	(Huang et al., 2023)
Tomato (*Solanum lycopersicum*)	CRISPR/Cas9	Cas9-gRNA construct	Segregation	NHEJ	SDN-1	*SlMlo1*	Powdery mildew resistance	(Nekrasov et al., 2017)
Soybean	CRISPR/Cas9	CRISPR-RNPs	Protoplast transfection	NHEJ	SDN-1	*GmCPR5*	DNA-free protoplast mutagenesis	(Subburaj et al., 2022)
Soybean (*Glycine max*)	CRISPR/Cas12a	Cas12a RNPs	PEG	NHEJ	SDN-1	*GmFAD2-1A/GmFAD2-1B*	DNA-free FAD2 mutagenesis	(Kim et al., 2017)
Soybean (*Glycine max*)	CRISPR/Cas9	Cas9-gRNA construct	Segregation	NHEJ	SDN-1	*GmFT2a*	Late-flowering mutants	(Cai et al., 2018)
Barley (*Hordeum vulgare)*	CRISPR/Cas9	Cas9-gRNA construct	Segregation	NHEJ	SDN-1	*HvLOXA/HvLOXB/HvLOXC1*	Improved grain storability	(Zeng et al., 2025)
Potato (*Solanum tuberosum*)	CRISPR/Cas9	CRISPR-RNPs	Protoplast transfection	NHEJ	SDN-1	*GBSS/GBSSI*	Amylose-free potato mutants	(Andersson et al., 2018)
Potato (*Solanum tuberosum*)	CRISPR/Cas9	Virus expressed Cas9 and gRNA	Replicon -based delivery	NHEJ	SDN-1	*StPPO2/StPPO1*	Reduced tuber browning	(Grbich et al., 2026)
Potato (*Solanum tuberosum*)	CRISPR-Cas9n	Base editing construct	Segregation	Base editing	SDN-2	*StALS1/StALS2*	Transgene-free herbicide resistance	(Veillet et al., 2019)
Potato (*Solanum tuberosum*)	CRISPR/Cas9	Cas9-gRNA construct	Selection of non-integrated plants	NHEJ	SDN-1	*PDS*	Transgene-free *PDS* mutants	(Bánfalvi et al., 2020)
Potato (*Solanum tuberosum*)	CRISPR/Cas12a	Cas12a construct	*Agrobacterium*-mediated transient co-editing	Base editing/NHEJ	SDN-1/SDN-2	*StALS/StDMR6*	Transgene-free *DMR6* mutants	(Huang et al., 2023)
Lettuce (*Lactuca domestica*)	CRISPR/Cas9	Cas9 RNP	Protoplast transfection	NHEJ	SDN-1	*LsBIN2*	DNA-free BIN2 mutants	(Woo et al., 2015)
Apple (*Malus domestica*)	CRISPR/Cas9	Cas9 RNP	Protoplast transfection	NHEJ	SDN-1	*DIPM-1/DIPM-2/DIPM-4*	DNA-free apple mutagenesis	(Malnoy et al., 2016)
Citrus (*Citrus sinensis*)	CRISPR/Cas9	Cas9 and gRNA (Transient DNA)	Short-term chemical selection after transient transformation	NHEJ	SDN-1	*CsPDS*	Transgene-free *PDS* mutants	(Li H et al., 2025)
Citrus (*Citrus sinensis* cv. Hamlin)	Cas12a + CBE	Cas12a and CBE construct (transient)	Cas12a/CBE co-editing (*ALS* selection + GFP screening)	NHEJ and base editing	SDN-1 and SDN-2	*CsALS/LOB1 promoter*	Canker-resistant Hamlin	(Jia et al., 2024)
Citrus	CRISPR/Cas12a	Cas12a RNPs	PEG	NHEJ	SDN-1	*CsLOB1*	Transgene-free *LOB1* mutants	(Su et al., 2024)
Citrus	CRISPR/nCas9	CBE	*Agrobacterium*-mediated co-editing	Positive/negative selection	SDN-2	*CsALS/CsNPR3*	Transgene-free co-edited citrus	(Rocha et al., 2025)
Citrus	CRISPR/Cas12a	Cas12a construct	*Agrobacterium*-mediated transient co-editing	Base editing/NHEJ	SDN-2/SDN-1	*CsALS/LOB1 promoter*	Transgene-free canker resistance	(Huang et al., 2023)
Grapevine (*Vitis vinifera*)	CRISPR/Cas9	Cas9 RNPs	Protoplast delivery	NHEJ	SDN-1	*VvMLO7*	DNA-free grapevine mutagenesis	(Malnoy et al., 2016)
Grapevine	CRISPR/Cas9	Cas9 RNP	Protoplast transfection	NHEJ	SDN-1	*GFP reporter*	Regenerated DNA-free plants	(Najafi et al., 2023)
Grapevine	CRISPR/Cas9	Cas9 RNP	Protoplast transfection	NHEJ	SDN-1	Not fixed/gene of interest	DNA-free editing protocol	(Bertini et al., 2025)
Grapevine	CRISPR/Cas9	Cas9 RNP	Protoplast transfection	NHEJ	SDN-1	*VviDMR6/VviMLO6*	Non-chimeric DNA-free mutants	(Scintilla et al., 2022)
Grapevine	CRISPR/Cas9	Cas9 RNP	Protoplast transfection	NHEJ	SDN-1	*VviDMR6-1/VviDMR6-2*	Reduced downy mildew susceptibility	(Giacomelli et al., 2024)
Brassica (*Brassica napus*)	CRISPR/Cas9	Cas9 RNP	Protoplast transfection	NHEJ	SDN-1	*FRI/PDS*	no mutations detected in B. napus	(Murovec et al., 2018)
Brassica	CRISPR/Cas9	Cas9 and mobile gRNA expressed in rootstock	Grafting based CRISPR mobility	NHEJ	SDN-1	*NIA1*	Graft-mobile heritable edits	(Yang et al., 2023)
Banana (*Musa* spp)	CRISPR/Cas9	Cas9-gRNA construct	Selection of non-integrated plants	NHEJ	SDN-1	*pds/LCYb*	Transgene-free Cavendish mutants	(Kato et al., 2025)
Banana	CRISPR-nCas9	Cas9-gRNA, CBE	Transient DNA expression	Base editing	SDN-2	*MaALS*	Transgene-free herbicide resistance	(Van Den Broeck et al., 2025)
Banana	CRISPR/Cas9	Cas9, dual gRNA, Reg-2 suicide cassette	Gene deletion system	NHEJ	SDN-1	*MaPDS*	Transgene-free albino mutants	(Hu C et al., 2023)
Carrot (*Daucus carota*)	CRISPR/Cas9	Cas9 RNP	Protoplast transfection	NHEJ	SDN-1	*Acid soluble invertase II*	Transgene-free carrot mutants	(Yarra and Krysan, 2025)
Carrot (*Daucus carota*)	CRISPR-nCas9	Cas9-gRNA, CBE	Protoplast transfection	Base editing	SDN-2	*CENH3*	Transgene-free base-edited carrot	(Meyer et al., 2022)
Cabbage	CRISPR-Cas9	Cas9-gRNA expressed in Haploid inducer	Hi-Edit	NHEJ	SDN-1	*BoNAL1/BoMYBL2*	Customized anthocyanin cabbage	(Li et al., 2025)
Melon (*Cucumis melo* L.)	CRISPR-Cas9	CRISPR-RNPs	In-planta bombardment (iPB-RNP)	NHEJ	SDN-1	*CmACO1*	Long shelf-life melon	(Sasaki et al., 2025)
Watermelon (*Citrullus lanatus*)	CRISPR-Cas9	Cas9-gRNA plasmid	Segregation	NHEJ	SDN-1	*ClSPL/SPOROCYTELESS*	Diploid seedless watermelon	(Jiang et al., 2024)
Tobacco (*Nicotiana tabacum*)	CRISPR/Cas12a	Cas12a construct	*Agrobacterium*-mediated transient co-editing	Base editing/NHEJ	SDN-2/SDN-1	*NtALS/NtPDS*	Transgene-free albino tobacco	Huang et al., 2023
Tobacco	CRISPR-Cas9	Cas9-gRNA plasmid	*Agrobacterium* transient transformation	NHEJ	SDN-1	*NtPDS*	Non-transgenic PDS mutants	(Chen et al., 2018a)
Poplar	CRISPR-nCas9	Cas9-gRNA, CBE	*Agrobacterium* transient transformation	Base editing	SDN-2	*ALS/CCoAOMT1*	Transgene-free base-edited poplar	(Hoengenaert et al., 2025)
Poplar	CRISPR/nCas9	CBE	*Agrobacterium*-mediated co-editing	Positive/negative selection	SDN-2	*ALS/Pt4CL1*	Transgene-free co-edited poplar	(Rocha et al., 2025)
Chickpea	CRISPR/Cas9	Cas9 RNP	Protoplast transfection	NHEJ	SDN-1	*4CL/RVE7*	DNA-free protoplast editing	(Badhan et al., 2021)
Sorghum	CRISPR-Cas9	Cas9-gRNA plasmid	Transient transformation via particle bombardment	NHEJ	SDN-1	*PDS*	Transgene-free albino sorghum	(Zhang et al., 2025)

Rice has been a flagship crop for transgene-free gene-editing because of the availability of protoplast and regeneration protocols. In many laboratories, a foundational demonstration of transgene-free gene-editing uses preassembled Cas9-gRNA RNPs, which are delivered to rice protoplasts to regenerate rice plants. For example, one of the earliest demonstrations showed direct delivery of CRISPR-RNPs into plant protoplasts (*Arabidopsis*, tobacco, lettuce, and rice), enabling efficient DNA-free gene-editing, and producing targeted mutations (Woo et al., 2015). Similarly, knockout of yield-related genes such as *GW2*, *GW5*, and *TGW6* significantly increased grain size and weight, and the authors demonstrated that transgenes can be segregated to produce transgene-free gene-edited rice plants (Li et al., 2016; Xu et al., 2016). Point mutations in the ALS gene conferred herbicide tolerance using RNP-mediated gene editing, yielding plants free of transgenic sequences (Zhang R et al., 2021).

Bread wheat (*Triticum aestivum*) has a complex hexaploid genome and has historically posed challenges for precise breeding. Several studies have demonstrated CRISPR-Cas-mediated transgene-free gene-editing in wheat for effective trait improvement using SDN-1 modifications, achieved through segregation or RNP-mediated delivery. Examples include transgene-free gene-editing approaches in wheat, further supported by the delivery of CRISPR RNPs into wheat protoplasts, enabling targeted mutagenesis of genes such as *TaPDS* and *TaALS* (Liang et al., 2017; Zhang et al., 2016). In another study, Hamada et al. (2018) demonstrated in planta, transgene-free gene-editing in wheat by biolistic delivery of CRISPR/Cas9 plasmids into the shoot apical meristem, achieving heritable *TaGASR* mutations (Hamada et al., 2018).

Maize has strong translational relevance for global food and feed systems, and a key breakthrough was the demonstration of CRISPR RNP delivery into maize embryo cells, followed by regeneration of plants with edited alleles (Svitashev et al., 2016). In this study, CRISPR RNP complexes were delivered into immature maize embryos by particle bombardment, generating targeted mutations at several loci (*ALS2, MS26, MS45*, and *Liguleless1*) without vector DNA, thereby producing edited plants free of transient effects and with reduced off-target effects compared to DNA delivery systems. In addition, transgene-free gene-edited maize lines with a useful agronomic semidwarf phenotype were obtained by editing the *GA20ox3* gene and segregating out the CRISPR cassette in later progenies (Zhang et al., 2020). A recent study reported transgene-free gene-editing in sorghum, an essential staple in arid and semi-arid regions. Zhang et al. (2025) demonstrated transgene-free gene-editing in sorghum within a single generation using particle bombardment targeting the PDS gene (Zhang et al., 2025).

Legumes and oilseed crops are central to protein and lipid security, but their transformation and regeneration are challenging. Transgene-free gene-editing in oilseed crops remains uneven, with most studies focusing on soybeans. For example, the flowering time gene *GMFT2A* was edited with CRISPR-Cas9, and homozygous T1-T2 transgene-free mutants with SDN-1 modifications were obtained after segregation of the CRISPR cassette (Cai et al., 2018). A recent study avoided DNA altogether and used in-planta bombardment of CRISPR-RNPs targeting the shoot apical meristem of the embryonic axis, enabling genotype-independent editing without introducing foreign DNA or using a tissue culture (Kuwabara et al., 2024). Similarly, CRISPR RNPs were used for DNA-free editing in soybean protoplasts for rapid knockout screens and target validation (Subburaj et al., 2022). Transgene-free gene-editing in chickpea is limited to protoplast editing due to the challenges of regeneration. A landmark study demonstrated DNA-free CRISPR RNP delivery into chickpea protoplasts, targeting drought-and stress-related loci (Badhan et al., 2021).

In horticultural crops, several studies have demonstrated that transgene-free gene-editing can improve disease resistance, shelf life, and quality. For example, transgene-free gene-editing in Brassica vegetables was achieved by PEG-mediated delivery of preassembled CRISPR RNPs targeting two genes (*FRI* and *PDS*) into *B. oleracea* and *B. rapa* protoplasts, yielding efficient, dose-dependent SDN-1 indel mutations in endogenous genes without any foreign DNA integration (Murovec et al., 2018). In another study, transgene-free gene-editing in lettuce was achieved by direct delivery of CRISPR-Cas RNPs, one of the earliest DNA-free examples for editing lettuce genes, producing targeted mutations without foreign DNA integration (Woo et al., 2015). DNA-free gene-editing in wild tetraploid tomato (*Solanum peruvianum*) was achieved using PEG-mediated delivery of CRISPR-Cas9 RNPs into regenerable protoplasts, generating heritable SDN-1 mutations in genes related to RNA silencing (*SpRDR6* and *SpSGS3*), pathogen response, and disease resistance without foreign DNA integration or chromosomal abnormalities (Lin et al., 2022). In fruit crops, the delivery of CRISPR RNPs into grape and apple protoplasts, targeting susceptibility genes linked to powdery mildew (grape) and fire blight (apple), demonstrated DNA-free gene-editing in woody fruit crops (Malnoy et al., 2016). Recently, a DNA-free editing protocol has been demonstrated in grapevine, thus extending transgene-free gene-editing in perennials (Najafi et al., 2023).

## 8 Commercially approved transgene-free gene-edited crops

CRISPR technology has been widely used for precise genetic modifications in both model and commercial crops, enabling practical agricultural innovations. For instance, in model plants, CRISPR has been widely applied to edit the *Arabidopsis* and rice genomes (Saini et al., 2023). These advances have facilitated functional genomics, base editing, and multiplex gene editing, yielding insights into complex traits and disease resistance. The findings from these model systems have subsequently been translated into commercial crops such as rice (Priyadarshini, 2025), wheat (Tajima et al., 2025), maize (Wang et al., 2022), and mushrooms (Waltz, 2016). Examples include rice with improved yield, disease resistance, and nitrogen use efficiency achieved through gene editing (Hu B et al., 2023). Similarly, CRISPR-based gene editing has been applied to maize to enhance drought tolerance (Wang C et al., 2025), while modifications in tomatoes have focused on increasing shelf life and nutritional value (Waltz, 2022). Several transgene-free gene-edited crops have been successfully commercialized, often passing the strict regulatory measures applied to GM crops (**Table 4**). For instance, non-browning mushrooms were among the first commercially available crops developed using CRISPR technology. Further commercial examples include high-GABA tomatoes produced *via* CRISPR and marketed in Japan, as well as waxy corn commercialized by Corteva Agriscience. Additionally, several other crops are currently awaiting regulatory approval, including disease-resistant and high-yielding rice and soybeans with enhanced healthy oil content. These developments collectively underscore the potential of CRISPR to generate precise gene-edited crops capable of addressing key challenges in agriculture and ensuring food security.

**Table 4:** Commercialized transgene-free gene-edited crops.

Crop	Target trait	Technology	Developer	Country	SDN	Transgene-free	Regulatory status	Key feature	Reference
Tomato	Enhanced gamma-aminobutyric acid (GABA) content	CRISPR-Cas9	Sanatech Seed	Japan	SDN-1	Yes	Commercialized 2021	Highlighted consumer acceptance of health-promoting transgene-free gene-edited food	(Waltz, 2022)
Waxy corn	High amylopectin content	CRISPR-Cas9	Corteva Agriscience	USA	SDN-1	Yes	Commercialized 2020	Demonstrated the commercialization of maize with an improved quality trait	(Wang et al., 2022)
Soybean	Healthier oil profile (high oleic acid, low linoleic)	TALENs/CRISPR	Calyxt/Cibus	USA	SDN-1	Yes	Commercialized 2019	Showed the commercial value of nutrition-focused gene editing	(Calyxt, 2019)
Mushroom	Non-browning	CRISPR-Cas9	Pennsylvania State University	USA	SDN-1	Yes	Commercialized 2016	Established an important regulatory precedent for transgene-free gene-edited SDN-1 crops	(Waltz, 2016)
Canola	Herbicide tolerance	ODM	Cibus	USA/Canada	SDN-1	Yes	Commercialized	Demonstrated the commercial potential of transgene-free gene-editing for improving oil quality	(Chhalliyil et al., 2020)
Rice	Enhanced yield and disease resistance	CRISPR-Cas9	CAAS	China	SDN-1/2	Yes	Biosafety certificate issued	Demonstrated the role of transgene-free gene-editing in food security and yield improvement	(Ortega et al., 2022)
Rice	Stress resistance	CRISPR-Cas9	IARI	India	SDN-1/2	Yes	Commercialized	Highlights the potential of CRISPR to enhance productivity and strengthen food security	(Priyadarshini, 2025)
Banana	Non-browning	CRISPR-Cas9	Tropic Biosciences	Philippine	SDN-1	Yes	Commercialized	Demonstrates the value of transgene-free gene-editing in vegetatively propagated crops	(Pittman et al., 2026)

## 9 Evolving regulatory frameworks of transgene-free gene-edited crops

**Figure 6:**
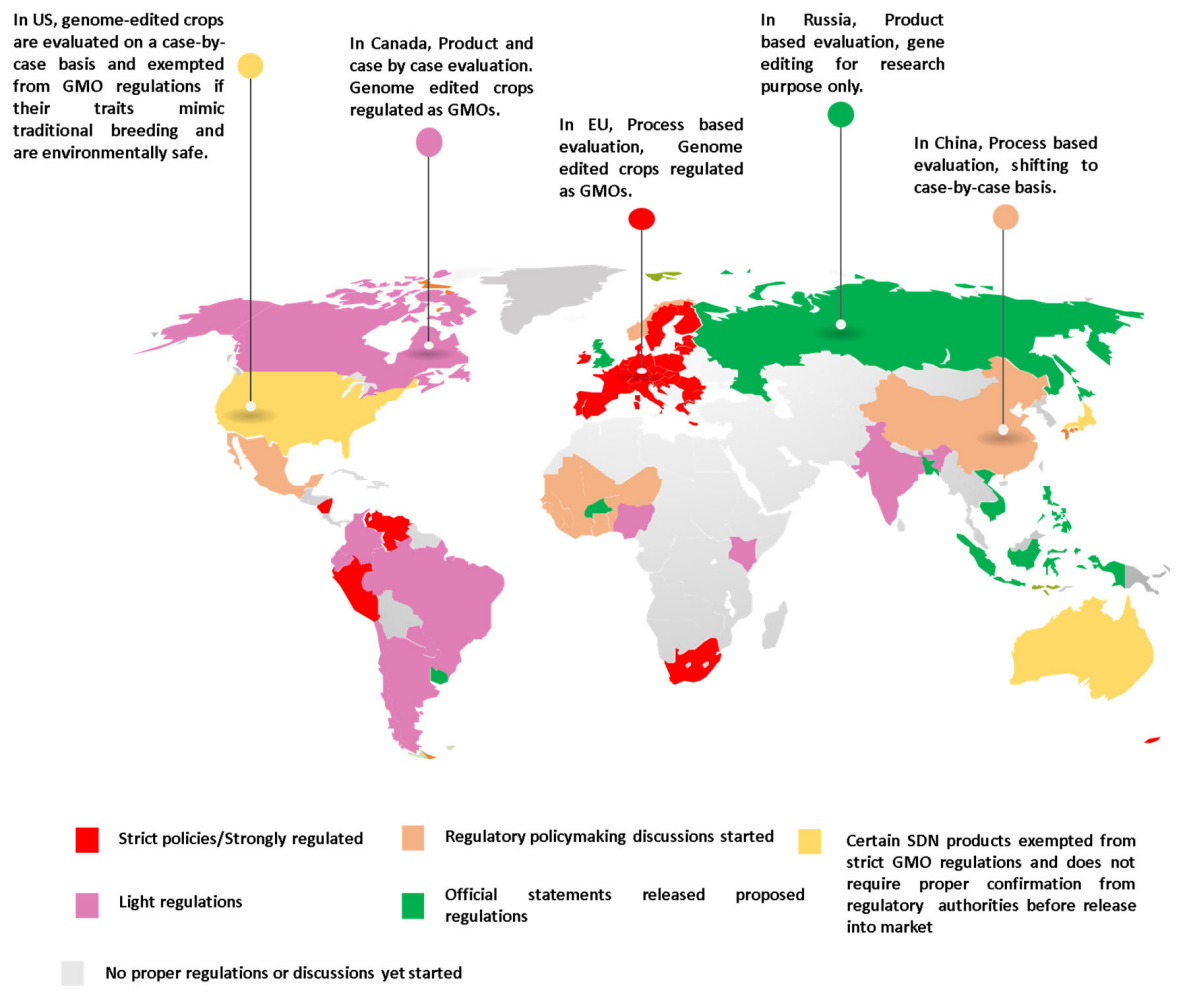
The status of genome-editing regulations worldwide.

Regulations for gene-edited crops, especially, remain among the strongest factors in agricultural biotechnology that will determine whether we can move from laboratory pipelines into farmers’ fields and international markets (Lassoued et al., 2019). Unlike transgenic GM crops, where recombinant DNA integration is central to regulatory triggers and risk assessment, SDN-1 and some SDN-2 outcomes often resemble alleles that could arise through conventional breeding or spontaneous mutations, challenging older regulatory frameworks (Ahmad et al., 2023; Podevin et al., 2013; Schiemann et al., 2020). Regulatory systems are diverging globally into i) product-based frameworks that exempt certain edits, ii) hybrid frameworks that maintain oversight but streamline pathways, and iii) a process-oriented framework that still treats all gene-edited plants (SDN-1/2/3) as GMOs, unless revised or new legislation is introduced (**Figure 6**). In the following section, we emphasize the global regulatory framework, with a particular focus on SDN-1 and SDN-2 modifications (**Table 5**).

**Table 5:** Global regulatory framework of transgene-free gene-edited crops.

Countries	Regulatory trigger for gene-edited crops	Law for regulating gene-edited crops/year	Regulatory authorities	SDN-1 (exempt or regulated as GMOs)	SDN-2 (exempt or regulated as GMOs)	SDN-3 (exempt or regulated as GMOs)	Labelling requirement	Transgene-free gene-edited crops approved	Critical analysis and key insights	References
USA	Product-based (USDA-APHIS SECURE exemption; EPA/FDA product-specific)	USDA-APHIS SECURE Rule (7CFR Part 340, finalized 2020, in effect 2021)	USDA-APHIS, EPA, FDA	Often exempt from APHIS if confirmed transgene-free	Often exempt if it meets the exemption criteria and no plant pest risk; confirmation must be available	Regulated as GE/GM under APHIS; review/permit may apply	No gene editing-specific mandatory labeling.	Multiple gene-edited crops have been approved for commercialization	• Product-based oversight that accelerates the innovation and commercialization of transgene-free edited crops • Complicates international regulatory alignment	(Hoffman, 2021; Ricroch et al., 2026; Tachikawa and Matsuo, 2023)
EU	Process-based (GMO framework)	Directive 2001/18/EC + Regulations (EC) 1829/2003 & 1830/2003; ECJ Case C-528/16 (25 July 2018)	European Commission, EFSA, Member State authorities	Regulated as a GMO under the current framework	Regulated as a GMO	Regulated as a GMO	GMO Labeling/traceability required under EU GMO rules	No	• Precautionary regulation limits the commercialization of all gene-edited crops • Gradual transition toward differentiated oversight through New Genomic Techniques (NGTs) proposals	(Andersen 2025a, b; Qin and Su, 2026; Ricroch et al., 2026)
Canada	Novel-trait/product-based (Plants with Novel Traits, PNT)	PNT framework; CFIA updated guidance (2023)	CFIA (PNT/environment/feed); Health Canada (food)	Regulated only if the trait is novel (PNT), otherwise considered conventional	Case by Case- Regulated if the trait is novel	Often regulated (PNT trigger applies; transgenic traits commonly novel)	No Mandatory labeling specific to gene-edited foods (general labeling rules apply)	Case-by-case commercialization is possible, if not PNT, or after assessment	• Novelty-based regulation provides flexibility but may create uncertainty • Trait-focused assessment supports science-based regulation of transgene-free gene-edited crops	(Lassoued et al., 2024; Lubieniechi et al., 2025; Ruder and Kandlikar, 2023)
Australia	Product/risk-based; SDN-1 is excluded from GMO regulations	Gene Technology Regulations amendment implemented on 8 October 2019	OGTR; FSANZ	Exempt (not GMO) if SDN-1 type and no foreign DNA present	Regulated as GMOs	Regulated as GMOs	GM labeling applies where the product is GM (FSANZ); SDN-1 is generally not GM-labeled	SDN-1 products are possible under exclusion; broader approvals are evolving	• Supports proportionate regulation for SDN-1 transgene-free gene-edited crops • Exemptions facilitate innovation while maintaining targeted biosafety oversight	(Jones et al., 2022; Thygesen, 2024)
New Zealand	Process-based (strict GMO)	HSNO Act; High Court decision (May 2014) classified ZFN-1/TALENs as GMO techniques	EPA New Zealand	Regulated as a GMO	Regulated as a GMO	Regulated as a GMO	GMO controls/labeling apply when relevant	No	• Strict GMO classification limits commercialization and innovation opportunities • Maintains one of the most precautionary gene-editing frameworks globally	(Jones et al., 2024; Thygesen, 2024; Vengadesen et al., 2025)
Argentina	Product-based/Case-by-case (transgene presence)	Resolution 173/2015 (case-by-case GMO determination for NBT products)	CONABIA	Typically exempt (non-GMO confirmation) if no transgene in the final product	Typically exempt if no foreign DNA in the final product	Regulated as a GMO	No Mandatory national labeling for GM/gene-edited foods	Several non-GMO determinations issued for gene-edited products	• Pioneer of proportionate regulatory oversight for transgene-free gene-edited crops • Early regulatory adoption accelerated innovation and technology deployment	(Ricroch et al., 2026; Goberna et al., 2024; Kuiken and Kuzma, 2021; Kumawat et al., 2024)
Brazil	Product-based/case-by-case	CTNBio Normative Resolution No. 16/2018 (published 15 January 2018)	CTNBio	Often exempt if no foreign DNA in the final product (case by case)	Often exempt if no foreign DNA in the final product (case by case)	Regulated as a GMO	GM labeling applies to GMOs; exempt products are generally not GM labeled	Non-GMO determinations issued	• Case-by-case regulation balances innovation and biosafety considerations • Supports commercialization through science-based regulatory decision-making	(Da Cunha et al., 2025; Kuiken and Kuzma, 2021)
Chile	Product-based; foreign DNA presence; case by case	SAG consultation/determination process (implemented 2017)	SAG (Servicio Agrícola y Ganadero)	Exempt/regulated as conventional if no foreign DNA	Exempt/regulated as conventional if no foreign DNA (case by case)	Regulated as a GMO	NO GM labeling if confirmed non-GMO (case by case)- GM Labeling required	Determination issued; commercialization evolving	• Early adopter supporting proportionate regulation of transgene-free gene-edited crops • Case-by-case regulation, promoting innovation while maintaining biosafety oversight	(Sánchez, 2024; Zarate et al., 2023)
Kenya	Product-based/case-by-case under Genome Editing Guidelines	National Biosafety Authority Genome Editing Guidelines (February 2020; revised 2025)	National Biosafety Authority (NBA)	May be exempt (non-GMO) if there is no foreign DNA in the final product	May be exempt (non-GMO) if no foreign DNA in the final product (case by case)	Regulated as a GMO under the Biosafety Act, 2009	GMO labeling applies to GMOs; exempt products are considered conventional	Confined field trial approvals reported	• Emerging regulations balance innovation, biosafety, and agricultural development • Demonstrates growing African adoption of genome-editing regulatory frameworks	(Akinbo et al., 2025; Amoah et al., 2024; Ongu et al., 2023)
South Africa	Process-based on the GMO Act	GMO Act No. 15 of 1997 (as amended)	Executive Council under the GMO Act; DALRRD and related bodies	Commonly treated as a GMO pending clear exclusion	Commonly treated as GMO	Regulated as a GMO	GMO labeling rules apply where mandated	No documented gene-edited crop approval. GM crop approval exists	• Strict GMO-based oversight limits gene-editing commercialization • Emphasizes biosafety assessment through cautious regulatory governance approaches	(Runo et al., 2024; Townsend and Shozi, 2021)
China	Dedicated gene editing pathway for plants without foreign DNA, otherwise a GMO framework	MARA Guidelines for Safety Evaluation of Gene-Edited Plants for Agricultural Use (Trial) issued 24 June 2022	MARA	Regulated under gene-edited guidelines pathway (scope: no foreign genes)	Regulated under gene-edited guidelines pathway (scope: no foreign genes)	Regulated as a GMO	GMO labeling applies to GMOs; gene-edited labeling not clearly mandated similarly	Gene-edited varieties received safety certificates/approval (reported December 2024)	• Increasing acceptance of transgene-free gene-edited crops, particularly with SDN-1 modifications • Regulatory reforms encourage innovation	(Fernández Ríos et al., 2025; Liang et al., 2022; Mallapaty, 2022; Ricroch et al., 2026; Yang et al., 2024).
India	Process based GMO Rules 1989 with SDN-1/SDN-2 (transgene-free) exemption	MoEF&CC Office Memorandom (30 March 2022) + DBT Genome Edited Plants Guidelines (May 2022)	MoEF&CC; DBT GAEC (for GM)	Exempt from key provisions of the Rules, 1989, when transgene-free	Exempt from key provisions of the Rules, 1989, when transgene-free	Regulated as a GMO under the Rules, 1989	GM labeling is governed by existing standards	Yes	• Relaxed SDN-1 and SDN-2 regulations supporting food security and innovation • Promotes crop improvement through proportionate oversight of transgene-free gene-edited crops	(Prasad and Chimata, 2023; Sharma et al., 2025; Priyadarshini, 2025)
Japan	Product-based (foreign DNA presence determines GMO-like treatment); notification system	MHLW policy for genome-edited foods (released 27^th^ March 2019)	MHLW; MAFF	NO regulated as GMO if no foreign DNA is present; notification/consultation	Generally similar if no foreign DNA remains; notification/consultation	Regulated as a GMO	GMO labeling applies to GMOs. There is no mandatory labeling specific to genome-edited foods	Yes	• Notification-based regulation enables the rapid deployment of transgene-free gene-edited food products • Simplified oversight accelerates commercialization and consumer acceptance	(Kondo and Taguchi, 2022; Ishii, 2025)
Philippine	Product-based/case-by-case via Plant Breeding Innovation (PBI)	DA Memorandum Circular No. 8, series of 2022 (PBI)	Philippine Department of Agriculture (DA)	May be exempt/not considered GE if evidence shows no foreign DNA/new genetic combination	Similar case-by-case determination	Regulated as GE/GMO	No GM-specific labeling applies	Regulatory pathways active; determination ongoing- gene dited Banana approved	• Emerging regulations support innovation, while biosafety frameworks continue to evolve • Demonstrates increasing regional acceptance of transgene-free gene-editing	(Jones et al., 2022; Tachikawa and Matsuo, 2023)
Thailand	New regulations approved during 2025	Genome Editing regulations approved in 2025	Thai agriculture/food regulators (per implementing rules)	Permitted under the new framework	Permitted under the new framework	Expected to face strict GMO-like regulations	GM labeling requirement. To be defined in revised regulations	Framework approved in 2025, approvals expected to follow	• Regulatory development is progressing cautiously • Moving toward science-based oversight supporting agricultural innovation	(Jones et al., 2022)
South Korea	Process-based (LMO Act); proposed revision covers gene editing as LMO	LMO Act; draft revision published 26^th^ May 2021	Multiple ministries; LMO Act system	Proposed treated as LMO, current approach cautious/LMO-oriented	Proposed treated as LMO, current approach cautious/LMO-oriented	Regulated as LMO/GMO	LMO/GM labeling rules apply where mandated	No major domestic cultivation; significant GMO import	• Regulatory uncertainty persists despite growing research and development investments • Gradually adapting oversight frameworks for transgene-free gene-edited agricultural products	(Jones et al., 2022; Yang and Zhou, 2024)
Pakistan	Evolving regulation: differentiated SOPs for SDN-1/SDN-2 vs SDN-3 indicated	National Seed Policy 2024 (SDN-1 & SDN-2 different SOPs); Biosafety amendments notified (2025 report)	National Biosafety Committee (NBC)	Regulated as a GMO. Policy indicates differentiated (potentially lighter) SOPs vs GMOs	Regulated as a GMO. Policy indicates differentiated (potentially lighter) SOPs vs GMOs	Regulated as a GMO	Existing GMO labeling/import controls; gene editing-specific labeling is not clear	GM cotton approved; gene-edited approvals not clearly documented	• The regulatory framework remains developing, potentially limiting commercialization • Transgene-free gene-editing offers significant potential for agricultural improvement	(Awais et al., 2024)
Saudi Arabia	GMOs regulated via national/GSO standards (import/labeling); gene editing not clearly separated	GSO-based technical regulations for GM foods/unprocessed agriculture products; national implementation	SFDA (food); related authorities	No clear gene editing exemption; classification likely case by case under existing rules	No clear gene editing exemption; classification likely case by case under existing rules	Regulated as a GMO	GSO-based GM labeling requirements apply (thresholds per applicable GSO standard)	No reported approvals for transgene-free gene-edited crops; GE crop activity is limited	• Regulatory policies evolving alongside national food security initiatives • Transgene-free gene-edited crops could support sustainable agriculture under Vision 2030	(Ahmad et al., 2021; Al-Mssallem et al., 2024)

### 9.1 United States: a product-oriented plant pest risk framework

The United States uses a coordinated framework involving the United States Department of Agriculture’s Animal and Plant Health Inspection Service (USDA-APHIS), the Food and Drug Administration (FDA), and the Environmental Protection Agency (EPA). Under 7 CFR part 340, APHIS focuses on plant pest risk rather than the method used to develop the crop. Under the SECURE (Sustainable, Ecological, Consistent, Uniform, Responsible, and Efficient) rule, many SDN-1 and SDN-2 modified crops are exempt from regulatory oversight if they do not contain foreign DNA (Hoffman, 2021; Ricroch et al., 2026). Developers can also request a Regulatory Status Review (RSR) to confirm exemption. As a result, many, particularly SDN-1-modified crops, qualify for deregulation or exemption, placing them outside APHIS oversight, especially when final outcomes could have been achieved through conventional breeding, and no plant-pest sequences are involved (Ahmad et al., 2024, 2023; Wolt and Wolf, 2018). SDN-2 modifications, however, are evaluated on a case-by-case basis, and transgene-free gene-edited SDN-2 crops may be exempt from strict regulatory oversight. In contrast, SDN-3 crops involving stable integration of the transgene are likely to trigger GMO regulatory review (Tachikawa and Matsuo, 2024). Approved for commercialization in the US are white button mushrooms (Waltz, 2016), waxy corn (Gao et al., 2020), and high-oleic soybean (Lee et al., 2025).

### 9.2 European union: process-based legal interpretation

In the European Union, gene-edited crops are regulated under a process-based framework, the most restrictive globally. According to the 2018 Court of Justice of the European Union ruling, all gene-edited crops, including SDN-1 and SDN-2, are regulated as GMOs under Directive 2001/18/EC (Vives-Vallés and Collonnier, 2019). SDN-3 crops are also regulated under conventional GM regulations (Sprink et al., 2016). As a result, all are currently subject to full GMO authorization, risk assessment, traceability, and labeling requirements, despite being transgene-free. This decision has been widely criticized for being scientifically inconsistent with the risk profiling of SDN-1 and for posing significant constraints on technological advancement and innovation. In response to these constraints and challenges posed by strict regulatory oversight, the European Commission proposed a new regulatory framework for New Genomic Technologies (NGTs), introducing a two-layered system in 2023 (Andersen, 2025a; Qin and Su, 2026). According to this proposal, certain NGT plants, especially SDN-1 and a limited number of SDN-2 plants lacking foreign DNA, will be exempted from the GMO authorization and labeling requirements. However, SDN-3 modifications will remain under strict GMO regulations (Andersen, 2025b). Until this legislation is adopted, all CRISPR crops remain under the GMO framework in the EU.

### 9.3 United Kingdom: post-brexit differentiation through precision breeding legislation

In 2022, England amended its GMO legislation through the Genetic Technology (Precision Breeding) Act 2023, establishing a new category for precision-bred organisms (PBOs), defined as organisms whose genetic modifications could have been introduced through natural selection or conventional breeding, distinguishing them from transgenic organisms. Under this framework, SDN-1 and some SDN-2 crops are not regulated as GMOs, while SDN-3 products involving insertions that cannot occur naturally do not fall under the PBO category and are subject to GMO oversight (Freeland et al., 2024; Tachikawa and Matsuo, 2023).

### 9.4 Japan: notification-based approach for SDN-1 edits

In 2019, Japan clarified that SDN-1 gene-edited crops, which involve small mutations without any foreign DNA, are not regulated as GMOs and require only prior notification rather than a full safety assessment (Kondo and Taguchi, 2022). According to the Ministry of Health, Labor and Welfare, gene-edited foods that do not contain any foreign genes are not treated as GMO products and are subject to a notification-based approach rather than pre-market risk assessment. This approach aligns closely with the deregulation of SDN-1 and many SDN-2 outcomes, provided developers confirm the absence of foreign DNA. In contrast, all SDN-3 outcomes with foreign DNA are subject to strict GMO regulations (Ishii, 2025). Transgene-free gene-edited tomato with improved GABA contents is approved for commercialization in Japan (Ishii, 2025; Waltz, 2022). Similarly, transgene-free gene-edited Red Sea Bream with increased muscle growth and Tiger Pufferfish with improved growth characteristics were also approved for commercialization (Kho et al., 2025).

### 9.5 China: differentiated oversight under state-controlled biosafety framework

The Ministry of Agricultural and Rural Affairs (MARA), in coordination with the Ministry of Ecology and Environment (MEE), oversees gene-edited crops (Mallapaty, 2022; Mu et al., 2025). In 2022, MARA issued guidelines for the safety and evaluation of gene-edited plants, formally distinguishing SDN-1 and certain SDN-2 outcomes with no foreign DNA from conventional GMOs and subjecting them to a simplified biosafety evaluation. SDN-3 products are still expected to undergo a stringent evaluation. China’s approach reflects a hybrid model that differentiates transgene-free gene-edited products from transgenic products while maintaining state oversight (Fernández Ríos et al., 2025; Liang et al., 2022; Ricroch et al., 2026; Yang et al., 2024). China has approved high-oleic gene-edited soybean (Wang R et al., 2025) and gene-edited wheat with powdery mildew resistance (Li et al., 2022; Mallapaty, 2022), for biosafety, both major steps toward commercialization. In addition, Chinese authorities have awarded biosafety certificates to gene-edited soybean, rice, wheat, and corn (Liang et al., 2025).

### 9.6 India: exemption with process-based framework

In India, transgene-free gene-edited crops are regulated under a process-based framework established by the 1989 Rules of the Environment (Protection) Act, with oversight by the Genetic Engineering Appraisal Committee (GEAC) under the Ministry of Environment, Forest, and Climate Change (Chimata and Bharti, 2019). In 2022, the Department of Biotechnology (DBT) issued guidelines stating that SDN-1 and SDN-2 transgene-free crops are exempt from certain provisions of the 1989 Rules governing GMOs, subject to defined molecular evidence requirements. SDN-3 modifications, however, are regulated as strict GMOs. This policy shift aligns India’s large agricultural sector with an innovative, evidence-based approach to crop genetic improvement (Prasad and Chimata, 2023; Sharma et al., 2025). Recently, India has approved CRISPR-edited transgene-free rice for commercialization (Priyadarshini, 2025).

### 9.7 Argentina: case-by-case administrative determination of transgene-free edited crops

Argentina’s regulatory framework is widely regarded as the most progressive for transgene-free crops (Goberna et al., 2024; Kuiken and Kuzma, 2021; Kumawat et al., 2024). Under Resolution No. 21/2021, developers and farmers may seek prior consultation to determine whether a product constitutes a GMO based on whether it contains a new combination of genetic material. Following prior consultation, SDN-1 and SDN-2 outcomes without a transgene are classified as non-GM (Ricroch et al., 2026; Vesprini et al., 2021; Whelan and Lema, 2019). This early-stage consultation model reduces uncertainty and has been influential internationally.

### 9.8 Brazil: normative resolution no.16 and administrative evaluation

Brazil’s National Technical Commission on Biosafety (CTNBio) provides regulatory oversight under Normative Resolution No. 16 (2018) (Da Cunha et al., 2025; Kuiken and Kuzma, 2021). Developers submit information to determine whether a product derived from gene-editing techniques will be regulated as GM or non-GM. All SDN-1 and SDN-2 products without foreign DNA are classified as non-GM and are subject to simpler regulatory oversight (Nepomuceno et al., 2020). All SDN-3 products are considered GMOs.

### 9.9 Australia: technique-based differentiation

Regulatory jurisdiction for transgene-free gene-edited crops lies with the Office of the Gene Technology Regulator (OGTR) under the Gene Technology Act 2000, while food safety is regulated by Food Standards Australia New Zealand (FSANZ) (Friedrichs et al., 2019; Tachikawa and Matsuo, 2023; Zhang et al., 2021). Amendments to the Gene Technology Act (2019) exempt SDN-1 techniques from GMO safety assessment when no repair template is used. As a result, SDN-1 modified crops are not subject to GMO approval; however, SDN-2 and SDN-3 remain regulated (Jones et al., 2022; Thygesen, 2024).

### 9.10 New Zealand: process-based classification

New Zealand maintains a strict, process-based regulatory framework established under the Hazardous Substances and New Organisms Act 1996. A 2014 High Court decision confirmed that gene editing constitutes genetic modification, thereby bringing all SDN-1, SDN-2, and SDN-3 modified crops under GMO regulation (Jones et al., 2024; Thygesen, 2024; Vengadesen et al., 2025). Although recent considerations recognize that certain “null segregants” may fall outside GMO control, the overall framework remains precautionary, and being transgene-free alone does not exempt gene-edited crops from GMO regulation.

### 9.11 Canada: trait-based “plants with novel trait (PNT)” framework

Canada is adopting a novel trait-based trigger to regulate gene-edited and transgene-free crops, regardless of the breeding method used. Plants developed through SDN-1 and SDN-2 modifications are assessed only if they express a PNT, regardless of whether foreign DNA is present. A PNT is any plant variety that expresses a trait, new to the Canadian environment, and that has the potential to affect the environment or human or animal health, regardless of whether it was developed through conventional breeding, transgenic methods, or gene editing. Regulatory jurisdiction lies with Health Canada for food safety and the Canadian Food Inspection Agency (CFIA) for environmental release (Hundleby and Harwood, 2022; Lubieniechi et al., 2025; Singer and Michaud, 2025; Smyth et al., 2020). Crops developed through SDN-1 and SDN-2 modifications are assessed only if they express a PNT, even if no transgene is present. If no risk is identified, they may be exempt from additional regulation.

### 9.12 Biosafety and risk assessment of transgene-free gene-edited crops

Transgene-free gene-edited crops are increasingly considered low-risk CRISPR products because they do not contain stably integrated foreign DNA in their genome. In addition, they often contain a precise few-nucleotide modification that is indistinguishable from naturally occurring mutations or those introduced through conventional breeding techniques. Nevertheless, the absence of foreign DNA does not automatically guarantee safety, and biosafety assessment remains an important component of responsible and transparent commercialization (EFSA, 2021). Potential biosafety concerns include off-target mutations, altered gene expression and metabolic profiles, genomic rearrangements at the target site, and trait-specific ecological effects, arising from the characteristics of the product rather than the gene-editing process itself (Kawall, 2019; Eckerstorfer et al., 2019, 2023). Advances in sequencing technologies (next-generation and whole-genome sequencing), AI and machine learning tools, and molecular characterization have improved the ability to confirm transgene-free and detect any off-target mutations in the final product. Current evidence indicates that well-characterized SDN-1 and most of the SDN-2 transgene-free gene-edited plants are likely to present risks comparable to those associated with plants generated through conventional breeding or mutation breeding process (Kawall, 2021; Eckerstorfer et al., 2023). Regulatory authorities are increasingly adopting a case-by-case, science-based risk evaluation for SDN-1- and SDN-2-modified plants that are free of foreign DNA in the genome. For example, the USA, Argentina, Japan, UK, and Brazil have adopted a relatively streamlined regulatory approach for transgene-free SDN-1 and SDN-2-edited plants that facilitates their assessment and commercialization. In contrast, the EU currently follows a more precautionary framework, although legislative proposals are under consideration to establish differentiated regulations for selected New Genomic Techniques (NGTs), particularly with modifications comparable to those of conventional breeding outcomes (Habets and Macnaghten, 2025; Kardung et al., 2026). Scientists believe that biosafety evaluation of transgene-free gene-edited plants should focus on the nature of the genetic modifications, the characteristics and intended use of the final product, and its environmental exposure, rather than solely on the process used to develop it. Such an evidence-based, scalable, and proportionate approach can protect human health and the environment, while promoting innovation, food security, and sustainable agricultural development.

## 10 Social, ethical, and public aspects of transgene-free gene-edited crops

Public perceptions and socio-economic outcomes will play a decisive role in whether transgene-free gene-edited crops can contribute to global food security and sustainable agriculture.

### 10.1 Ethical aspects and risk communication of transgene-free gene-edited crops

Ethical aspects of transgene-free gene-edited crops are very important, along with their legal status, social acceptance, intellectual property rights, and environmental responsibility (Munawar et al., 2024; Nouman Tahir and Zahra, 2025; R. Caradus and A. Turner, 2026). One current ethical issue concerns the intentional editing of genomes, which some stakeholders believe is unethical regardless of whether foreign DNA is present. Such beliefs are rooted in cultural, philosophical, or religious values and cannot be addressed solely through molecular arguments (Koloi‐Keaikitse et al., 2025). A second ethical issue concerns equal access and the distribution of benefits and burdens (Bansal and Kaur, 2025). For example, innovations and benefits may concentrate among large-scale industrial farming, seed companies, and large multinational agricultural companies such as Monsanto, Bayer, and Corteva, which may face resistance, even if there are no biological risks posed by transgene-free gene-edited crops (Abergel, 2024; Pray and Birner, 2025).

Traits that contribute significantly to broader societal impact, such as improved nutrition, reduced chemical inputs, climate change mitigation, and environmental benefits, may be ethically favored. Environmental protection and sustainable agriculture are therefore central to ethical considerations, while practices that intensify input dependence may face controversies similar to those associated with GM crops (Kole et al., 2025). Risk communication is another critical ethical concern for transgene-free gene-edited crops. For example, effective communication depends on product outcomes rather than on process, explaining what was done and why, which techniques were used, and the potential implications for health, agriculture, and the environment (Kato-Nitta et al., 2023; Paudel et al., 2023). Overstating precision without acknowledging risks can undermine credibility. A more trusted approach acknowledges uncertainty and explains how risk assessment was performed before editing using AI tools and managed through sequencing, phenotypic analysis, and comparative analysis after generating the product (Ruder and Kandlikar, 2023).

Engaging all stakeholders, including scientists, farmers, the public, policymakers, and regulatory and political authorities, is essential to foster trust and transparency and to avoid communication gaps. AI tools can help achieve these goals in the future. Recently, the regulatory framework allowed transgene-free gene-edited products to enter the market without public disclosure or tracking (Kuzma, 2023). Therefore, a responsible disclosure framework for transgene-free crops should clearly state the editing platform (e.g., Cas9, Cas12, base editing, or prime editing), the target gene and its biological functions, and the precise genetic modification introduced, including the size and location of indels. It should explicitly confirm the presence or absence of the transgene and describe the off-target assessment strategy, including sequencing depth and validation method. Moreover, the regulatory classification (SDN-1, SDN-2, and SDN-3) and the intended agronomic trait must be reported transparently to provide context for risks and benefits.

### 10.2 Consumer acceptance of transgene-free gene-edited crops

Consumer acceptance of gene-edited crops, particularly transgene-free gene-edited crops, is shaped by multiple factors, including technical risk assessment, perceived benefits, trust and transparency, and perceptions of naturalness. Multiple studies have demonstrated that no detectable off-targets were observed in gene-edited plants analyzed (Ballco et al., 2026; Shew et al., 2018). Moreover, transient and RNP-mediated delivery of CRISPR components further reduces the risks of any unintended editing in the final product (Ramakrishnan et al., 2025). Similarly, empirical studies have demonstrated that transgene-free gene-edited crops are perceived more favorably than transgenic GM foods (Shew et al., 2018; Spök et al., 2022). Acceptance of transgene-free gene-edited crops also varies by geography, socio-demographic factors, product type, and farming benefits (Dutta and Dutta, 2025). Another factor of acceptance is who benefits.

Consumers are more willing to accept gene-edited crops that deliver public and consumer benefits, such as reduced pesticide use, improved yields and nutritional quality, and environmental sustainability (Bearth et al., 2024; Götz et al., 2022). Conversely, acceptance declines for traits that benefit only producers or larger companies. This suggests that transgene-free gene-editing alone is insufficient to secure public acceptance; perceived societal values remain central. Similarly, trust in regulatory authorities and institutions strongly predicts consumer acceptance. When regulatory oversights and risk assessment remain transparent and credible, the public is more willing to accept transgene-free gene-edited crops as a continuation of conventional crops. When institutional trust is low, claims of transgene-free gene-editing, precision, and natural equivalence may be perceived as messaging rather than assurance.

### 10.3 Transgene-free gene-edited crops and developing economies

Transgene-free gene-edited crops hold significant potential to deliver benefits to smallholder farmers and developing countries, particularly in regions where agriculture is highly exposed to biotic and abiotic stresses (Kumar et al., 2023; Pixley et al., 2022). Evidence from transgenic GM crop adoption suggests that productivity-enhancing traits can, on average, increase yields and reduce insecticide use, though outcomes vary widely across contexts (Kumar et al., 2020). Transgene-free CRISPR crops may yield comparable benefits if traits that address locally relevant constraints are integrated into appropriate agronomic systems; however, many of these benefits are not automatic. Seed access, affordability, and licensing conditions remain decisive. Restrictive intellectual property arrangements, such as higher seed prices, could limit adoption or shift value away from producers. Regulatory and market factors also shape outcomes. Developing countries may lack capacity for molecular characterization, regulatory review, and post-release monitoring, leading either to overregulation that stalls innovation or to undersized oversight that undermines trust and trade compatibility (Adeel and Jones, 2024). Export-oriented systems face additional challenges if trading partners apply different regulatory definitions to genome-edited crops.

### 10.4 Intellectual property rights and access to CRISPR technologies

Intellectual property rights (IPR) are a major determinant of who can deploy CRISPR technologies and for what purpose. The CRISPR patent landscape is complex, encompassing foundational patents, delivery methods, and trade label claims, creating layered freedom-to-operate challenges for developers, particularly in the public sector (Arif et al., 2024). Even when regulatory barriers are lower for transgene-free gene-edited crops, additional intellectual property constraints can limit participation by smaller institutes and training programs in developing countries (Adeel and Jones, 2024). In addition to patents, the costs of generating regulatory data, conducting field trials, performing molecular characterization, and conducting compositional analysis can serve as a de facto barrier to market entry, reinforcing the concentration of innovation among well-resourced companies (Menz et al., 2020; Molinari et al., 2024). To address these challenges, several access strategies have been proposed, including humanitarian licensing for food security trades, patent pools for clearing houses, and public-private partnerships that preserve local breeding rights (Contreras, 2024; Upreti and Guida, 2025). Open-access tools for gRNA design and a shared transformation platform can further reduce barriers. Ultimately, the socio-economic contribution of transgene-free edited crops depends not only on their technical properties but also on governance choices that shape access, benefit-sharing, and trust. Without attention to these dimensions, gene editing risks reproducing early inequalities rather than fulfilling its promise for global food security.

## 11 The convergence of AI and CRISPR: OpenCRISPR-1 and DNA-guided Cas12a

AI and emerging digital technologies are rapidly reshaping the design, execution, and evaluation of transgene-free gene-editing developments by improving predictability and efficacy across the editing pipeline, from target discovery to field performance (Dixit et al., 2024; Patel et al., 2025). AI enables more reliable and scalable gene-editing strategies aligned with food security goals. For example, one of the most immediate contributions of AI to CRISPR technology lies in gRNA design and off-target predictions (Sahoo et al., 2026). More recently, AI models have focused not only on predicting cleavage, but also on editing outcomes, such as Indel spectra and frame-shift likelihood, which is particularly valuable for transgene-free workflows where transient delivery limits repeated selection cycles (Chia et al., 2025). OpenCRISPR-1 represents the first fully AI-designed Cas9-like gene editor, generated using large language models trained on extensive CRISPR-Cas sequence data. It demonstrates high editing efficiency with reduced off-target activity in human cells, highlighting the potential of *de novo* protein design to expand the CRISPR-Cas toolbox for precision gene editing and cell therapy applications. Building on the AI-designed nuclease OpenCRISPR-1, Das et al. developed a plant-optimized AI-designed editor (PAiD) and demonstrated that this synthetic nuclease supports efficient NHEJ-mediated knockout, adenine and cytosine base editing, and prime editing in rice, with performance comparable to or exceeding SpCas9 at multiple loci. These results establish that AI-designed nucleases are fully compatible with advanced plant gene-editing modalities and highlight their potential to expand the CRISPR toolbox beyond naturally evolved systems (Das et al., 2026).

Recently, Orosco et al. (2026) demonstrated that a synthetic DNA-based guide termed pseudo-guide DNA enables Cas12 to target RNA rather than DNA, thereby overcoming the traditional requirement for RNA guides and extending Cas12’s applications beyond genome editing. The authors demonstrated that the AsCas12a-ΨDNA system enables effective RNA detection, endogenous RNA knockdown, multiplex RNA regulation, and simultaneous DNA and RNA editing using a single CRISPR effector. Compared with RfxCas13d, the AsCas12a-ΨDNA exhibited significantly lower off-target effects in the tested cells. The study further demonstrated its therapeutic and diagnostic potential by achieving 100% diagnostic accuracy for the hepatitis C virus. The study also showed programmable RNA manipulation via the AsCas12a-ΨDNA by fusing it with RNAase H1 for RNA degradation and METTL3 for epi-transcriptome editing (Orosco et al., 2026).

## 12 Challenges and bottlenecks in transgene-free gene-edited crops

Despite rapid advances in CRISPR technologies and growing regulatory acceptance of transgene-free gene-editing outcomes, several technical challenges and research gaps continue to limit the broad deployment of CRISPR across crops and agroecological contexts. Addressing these challenges is essential to translate laboratory success into durable field-level impact and to ensure that transgene-free gene-editing contributes significantly to global food security (Kole et al., 2025; Sen et al., 2026). For example, a fundamental limitation of transgene-free gene-editing is the continued reliance on efficient delivery of CRISPR cargoes and plant regeneration systems, which remain highly species- and genotype-dependent (Bhattacharjee et al., 2023; He et al., 2018). Many crops, such as cotton, legumes, and woody perennials, are transformation recalcitrant (Nivya and Shah, 2023).

Editing polyploid crops (e.g., wheat, potato, cotton, sugarcane) and perennial species (date palm, grapevine, and fruit trees) introduces additional complexity and challenges for transgene-free gene-editing (Qin et al., 2025). In polyploids, functional redundancy among homologous gene copies often requires simultaneous editing of multiple alleles to achieve a measurable phenotype. Perennial crops pose additional challenges due to their long generation time and extended juvenile phase, which reduces the likelihood of removing the transgene cassette through segregation (Prado et al., 2023). For these species, direct transgene-free gene-editing is essential. Although proof-of-concept studies have been demonstrated in perennials, including grapevine and apple, regeneration efficiencies remain low (Ren et al., 2024; Schröpfer et al., 2022). Research gaps include optimizing multiplex transgene-free gene-editing using RNPs, improving allele-specific editing in polyploids, and developing in planta or regeneration-free systems. Advances in AI and deep learning for designing specific gRNAs, improving genome assembly quality in complex genomes, and developing miniature CRISPR-Cas systems with high precision will be critical for transgene-free gene-editing in perennials.

## 13 Future perspectives

Future progress in transgene-free gene-editing will be driven by next-generation CRISPR systems with improved precision that go beyond classical SDN-1 approaches. For example, base editing and prime editing are expected to play an increasingly important role by enabling precise nucleotide substitutions and small insertions without inducing DSBs, thereby minimizing off-target effects. Moreover, smaller Cas variants with relaxed PAM requirements, higher specificity, and compatibility with transient delivery will expand the scope of CRISPR in complex crop genomes. Delivery systems are equally important in transgene-free gene-editing, including nanomaterial-based transport, virus-mediated delivery, expression systems, and meristem- or pollen-mediated gene editing. These approaches can bypass the lengthy tissue culture process required for transforming recalcitrant crops.

Moreover, integrating AI tools to predict target sites, repair pathways, and outcomes will further improve the efficiency, reliability, and reproducibility of transgene-free gene-editing in crops. Democratizing CRISPR techniques, moving them beyond a few resourceful companies, is also necessary to fulfill their promise and potential for global food security. Democratizing CRISPR requires lowering technical, regulatory, and intellectual property barriers, particularly for public-sector breeding programs. Regulating and classifying transgene-free gene-edited crops will also play a critical role in their future and in global food security. A division of global regulatory frameworks for transgene-free gene-edited crops will lead to confusion, limited public acceptance, and restrictions on future innovation. A universal, scalable, and product-based risk-assessment regulatory framework will play a central role in the commercialization, safety, and public acceptance of these crops.
